# Changes in Protein Expression of Renal Drug Transporters and Drug‐Metabolizing Enzymes in Autosomal Dominant Polycystic Kidney Disease Patients

**DOI:** 10.1002/cpt.3715

**Published:** 2025-05-15

**Authors:** Annika C. Tillmann, Dorien J. M. Peters, Amin Rostami‐Hodjegan, Patricia Wilson, Jill Norman, Jill Barber, Zubida M. Al‐Majdoub

**Affiliations:** ^1^ Centre for Applied Pharmacokinetic Research, University of Manchester Manchester UK; ^2^ Department of Human Genetics Leiden University Medical Center Leiden Netherlands; ^3^ Certara Predictive Technologies (CPT) Sheffield UK; ^4^ UCL Centre for Kidney and Bladder Health, University College London (UCL) Royal Free Hospital London UK; ^5^ PKD Charity UK London UK

## Abstract

Autosomal dominant polycystic kidney disease is the most prevalent inherited kidney disease and leads to bilateral kidney enlargement and progressive loss of renal function, often over decades. Comorbidities include hypertension, flank pain, and bacterial infections. The condition often necessitates prolonged multidrug therapy. Given the kidneys' critical role in drug excretion, the progressive functional impairment in the disease can lead to complications such as drug overdosing and unexpected levels of drug–drug interactions. Studies of drug‐metabolizing enzyme and transporter expression in this patient group remain scarce. We conducted comprehensive global liquid chromatography–tandem mass spectrometry proteomic analyses of microsomal and cytosolic fractions from early‐stage (chronic kidney disease stage: 13, *n* = 16) and end‐stage autosomal dominant polycystic kidney disease patients (chronic kidney disease stage: 5, *n* = 14), comparing them with age‐matched healthy controls (*n* = 11). In the early‐stage ADPKD samples, most drug‐metabolizing enzymes and drug transporters did not differ significantly from the healthy controls. Exceptions were EPHX2 and SULT1C2 in the cytosolic fraction, which showed a more than 2‐fold decrease in abundance (*P* < 0.05). In contrast, the end‐stage ADPKD kidney samples showed a decrease in the abundance of most measured proteins. Several drug‐metabolizing enzymes, including CYP4F2, UGT1A6, UGT1A9, and UGT2B7, exhibited statistically significant reductions (*P <* 0.05). Among the drug transporters, OAT1, OAT3, and OCT2 were below the limit of quantification in most ES‐ADPKD samples. MDR1 was the only efflux drug transporter consistently measured, with an average abundance of 1.24 pmol/mg microsomal protein across all samples.

**Study Highlights**

**WHAT IS THE CURRENT KNOWLEDGE ON THE TOPIC?**

The mechanism by which autosomal dominant polycystic kidney disease (ADPKD) affects the renal expression of drug‐metabolizing enzymes and drug transporters remains unknown. Solely, MRP3 has been quantified in human renal cystic tissue.

**WHAT QUESTION DID THIS STUDY ADDRESS?**

The protein abundance of drug‐metabolizing enzymes and transporters was measured in early‐stage ADPKD, end‐stage ADPKD, and healthy control kidneys. The protein abundance levels were compared between the 3 groups.

**WHAT DOES THIS STUDY ADD TO OUR KNOWLEDGE?**

This study is the first to provide renal abundance data on drug‐metabolizing enzymes and transporters for ADPKD patients.

**HOW MIGHT THIS CHANGE CLINICAL PHARMACOLOGY OR TRANSLATIONAL SCIENCE?**

The study highlights the decrease in the abundance of a few specific drug‐metabolizing enzymes in early‐stage ADPKD kidneys and the overall decreased abundance of pharmacokinetic proteins in end‐stage ADPKD kidneys. These insights may impact drug dosing. They represent a crucial step toward accurate predictions of pharmacokinetics in ADPKD.

Autosomal Dominant Polycystic Kidney Disease (ADPKD) is a hereditary condition that affects up to 12 million people worldwide.^1^ It causes renal cyst formation, progressive bilateral kidney enlargement, and renal functional decline.^1^ Systemic manifestations include hepatic cysts, hypertension, bacterial infections, and flank pain. Most ADPKD patients require long‐term multidrug treatment.^1^, ^2^, ^3^, ^4^, ^5^

The kidneys play a significant role in the excretion of xenobiotics through glomerular filtration rate (GFR), active secretion, and reabsorption through drug transporters.^6^ The kidneys express several drug‐metabolizing enzymes (DMEs), including cytochrome P450 enzymes (CYP), UDP‐glucuronosyltransferases (UGT), flavin‐containing monooxygenases (FMO), and sulfotransferases (SULT).^7^, ^8^, ^9^, ^10^ In ADPKD, the renal functional decline may impact how drugs are processed and eliminated. Destruction of glomeruli and normal nephrons by renal cyst expansion, interstitial fibrosis, and chronic inflammation may alter the expression and function of drug‐metabolizing enzymes and transporters (DMET), thereby impacting drug disposition in ADPKD patients.^11^, ^12^, ^13^, ^14^ Yet, changes in renal DMET protein expression remain poorly understood. Doses of renally excreted drugs are adjusted to account for the reduced GFR.^2^, ^15^, ^16^ This assumes that the reduction in active secretion correlates with the decline in GFR. However, findings in chronic kidney disease (CKD) suggest that changes in the activity of several renal transporters are not simply related to GFR decline.^17^

CKD is an umbrella term for diseases that lead to renal function decline, including ADPKD.^18^ However, ADPKD distinguishes itself from other causes of CKD by renal enlargement, the formation of multiple renal tubular cysts, early onset, and a relatively slow progression rate, although there is a wide variability, dependent in part on the nature of the *PKD1* or *PKD2* mutation.^19^, ^20^ CKD study findings might not apply to ADPKD. However, they can serve as indicators.

Renal impairment can alter hepatic metabolism and drug elimination, though the extent of its clinical relevance depends on the drug and the severity of renal dysfunction. However, the systemic accumulation of xenobiotics resulting from impaired renal and hepatic elimination can significantly impact the overall drug disposition, potentially leading to altered pharmacokinetics and drug efficacy. Furthermore, decreased elimination might lead to minor drug–drug interactions (DDIs) becoming clinically relevant.^21^

Changes in the expression of renal DMET proteins will affect not only drugs primarily eliminated by the kidneys but also those metabolized elsewhere, including the liver. In ADPKD patients, such alterations in DMET expression and renal function may compromise drug clearance, metabolism, and overall effectiveness of any drug. Understanding changes in DMET protein expression allows the prediction of increased sensitivity to DDI using physiologically based pharmacokinetic (PBPK) models, even without clinical pharmacokinetic data. These projections can be verified using real‐world clinical data.^22^, ^23^ Thus, understanding the changes in the abundance of DMET proteins in the kidneys of ADPKD patients will help determine many aspects of dose optimization. Currently, MRP3 is the only drug transporter quantified in human ADPKD kidneys.^24^

Previous articles have used tissue samples from ADPKD kidneys to investigate the histological changes. The early‐stage (E‐) ADPKD samples still contained functional tissue, but functional tubular cells tended to be enlarged.^25^, ^26^, ^27^ This enlargement may be linked to dysregulated secretion of growth factors and cytokines in the disease.^11^ The E‐ADPKD cysts were of varying sizes and interwoven with functional tissue. Their origin was still discernible. In the end‐stage (ES‐) ‐ADPKD kidneys, only a few functional glomeruli remained. The origin of the cysts could no longer be determined. The tissue consisted of fibrotic tissue, cysts, and enlarged areas.^25^, ^26^, ^27^ Our study quantified the expression of DMET proteins in human E‐ ADPKD, ES‐ADPKD, and healthy control renal tissues using global liquid chromatography–tandem mass spectrometry (LC–MS/MS) proteomics and applying the HiN approach, with yeast alcohol dehydrogenase as the internal standard.^28^, ^29^ Since we aimed to quantify even low‐abundance DMET proteins, we fractionated the samples into cytosolic and microsomal fractions to enhance microsome enrichment. This represents the first proteomic analysis of a wide range of DMET proteins in renal human ADPKD tissue samples.

## METHODS

### Human kidney samples

The human kidney samples were provided from the PKD Charity‐sponsored PKD Biobank at UCL/ Royal Free London NHS Foundation Trust Hospital, London, UK (Collected in US, Ethical agreement number 20772). The samples stemmed from early‐stage (E‐) ADPKD (*n* = 16, CKD stage: 1–3, age: 19–63 years), end‐stage (ES‐) ADPKD (*n* = 14, CKD stage: 5, age:31–75 years) kidneys and healthy controls (*n* = 11, age: 15–75 years). Healthy controls were kidneys rejected for transplantation due to abnormalities in blood vessel structure, rendering surgery too challenging. The healthy control tissues showed no abnormalities in renal morphology. The samples were flash‐frozen in liquid nitrogen at the source and stored at −70°C. Demographic data of the donors are listed in the (**Table** **S1**).

### Kidney sample preparation

The tissue samples used in this study were selected to contain similar amounts of medulla and cortex. The samples were lysed in homogenization buffer using a mechanical homogenizer (Thermo Fisher Scientific, UK). The homogenates from each sample were separated into cytosolic and microsomal fractions and stored at −80° C. Each fraction (70 μg) was spiked with yeast alcohol dehydrogenase (0.056 μg) and digested using the filter‐aided sample preparation protocol.^28^, ^30^ The method is described in **Supplementary Data** (Methods, Sample preparation, Sections 13).

### Mass spectrometry analysis

The samples were reconstituted in 35 μL of loading buffer (3% ACN and 0.1% formic acid (FA) in HLPC water) and prepared for analysis with the UltiMate 3,000 rapid separation liquid chromatography (RSLC, Dionex, Surrey, UK) and the Q Exactive HF Hybrid Quadrupole‐Orbitrap mass spectrometer (Thermo Fisher Scientific, Bremen, Germany). Details on the LC–MS/MS analysis can be found in **Supplementary Data** (Methods, Section 4, Data analysis, Section 1).

### Data analysis and protein quantification

The raw data were analyzed with MaxQuant version 2.2.3.0 (Max Planck Institute, Martinsried, Germany). For the MaxQuant search, a modified database containing 71,790 proteins, including the standards added to the sample, was used.

The results obtained from MaxQuant were analyzed using a customized database based on the Uniprot Human Protein fasta file (https://www.uniprot.org/proteomes/UP000005640) with non‐human standards added. This database contains data from 21,520 proteins and is termed CAPKR11. Proteins not found in CAPKR11 were analyzed using the MaxQuant database 2020, which contains 71,782 proteins. A razor was created as described in our previous publication.^31^ The **Supplementary Data** (Methods, Data analysis, Section 1) provides details.

### Reproducibility of data

Similarities between technical replicates (E‐ADPKD: 4 replicates, ES‐ADPKD and Healthy controls: 3 replicates) were assessed by calculating the Percentage Identical Peptides (PIP). The inter‐sample correlation was evaluated by calculating the PIP and Percentage Identical Proteins (PIPr). A principal component analysis (PCA) was performed based on PIPr values to assess any patterns or clusters in the quantifiable protein appearances across different samples.^32^

### Statistical data analysis

Statistical analysis was conducted with GraphPad Prism 9.3.1 (La Jolla, CA). Kruskal Wallis test and a post‐hoc Dunn's test, which accounted for the multiple comparisons between the groups, were used.^33^ Changes were deemed statistically significant if the adjusted *P*‐value was below 0.05. **Supplementary Data** (Methods, Data Analysis, Section 5) describes statistical analysis.

## RESULTS

Our study analyzed human kidney samples from early‐stage (E‐) ADPKD (*n* = 16, CKD stage = 1–3) and end‐stage (ES‐) ADPKD samples (*n* = 14, CKD stage = 5) with age‐matched healthy controls (*n* = 11). Isolation of non‐cystic tissue was not feasible, as macroscopic and microscopic cysts were indistinguishable. The presence of cysts in ADPKD tissue made it challenging to differentiate between cortex and medulla. To address this, we investigated the abundances of cortical and medullary marker proteins, with the corresponding data shown in **Supplementary Data** (Results, Section 8). The microsomal and cytosolic fractions were isolated from human kidney tissues and analyzed with LC–MS/MS. Microsomal and cytosolic marker proteins were quantified to investigate the quality of the sample fractionation, as shown in **Supplementary Data** (Results, Section 5). All samples' total proteome MS signal intensities are documented in **Supplementary Data** (Results, Section 1 and **Table** **S2**).

Technical duplicates of randomly selected samples (*n* = 20) from cytosolic and microsomal fractions were run as quality control. The percentage identical peptide (PIP) values of the replicates are reported in the **Supplementary Data** (Results, Section 2 and **Table** **S3**). In total, 52,359 peptides were detected in the microsomal fractions and 48,820 in the cytosolic fractions. In all samples,’ microsomal and cytosolic fractions, 4,798 proteins were‐ quantified. On average, 1721 proteins were quantified in each sample. The ESADPD fractions consistently showed lower total protein amounts and lower number of proteins. Due to the limited availability of CKD stage 1–3 ADPKD kidney samples, these stages were grouped as the EADPKD group (*n* = 16).

The **Supplementary Data** provides detailed tables of PIP and PIPr values between the different samples (Results, Section 3, **Tables** **S4** **and** **S5**). Based on the PIPr values, a principal component analysis was conducted which can be found in the **Supplementary Data** (Results, Section 4, **Figure** **S1**).

### Comparison of abundance of drug‐metabolizing enzymes and drug transporters (DMETs) in the microsomal fraction

Our comparisons of DMET abundances across CKD stages 1 to 3 showed that in E‐ADPKD, disease progression did not impact enzyme and drug transporter abundance. In fact, for several proteins, the highest abundance was measured in CKD stage 3 samples. However, the number of ADPKD samples in CKD stage 1 and 2 is limited. It is possible that a trend of a stepwise decrease from CKD stage 1 to 3 could be observed with a higher number of tissue samples.

#### Drug‐metabolizing enzymes (DMEs)

**Figure 1:**
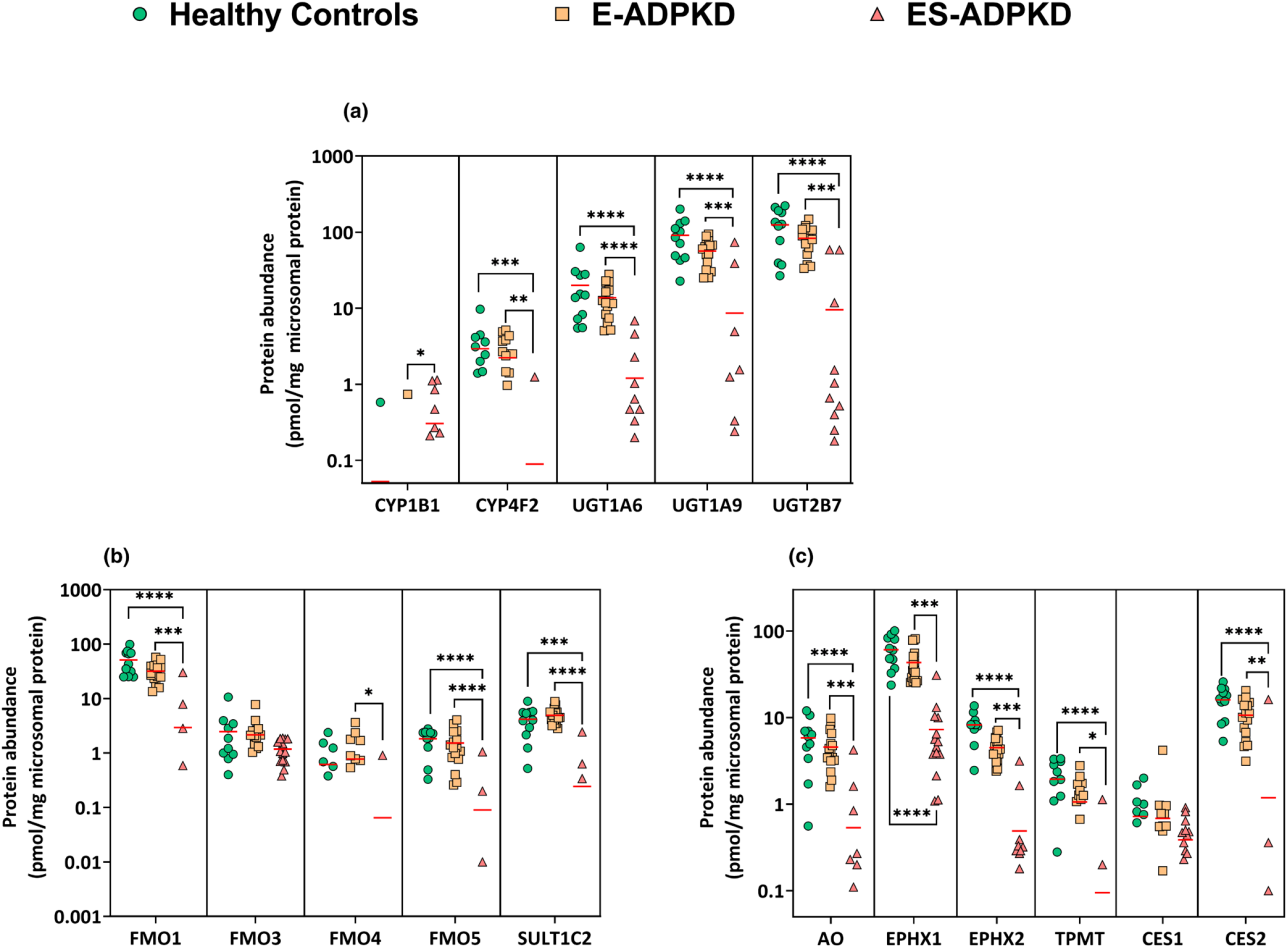
Total abundance of CYP and UGT enzymes (**a**), FMOs and a SULT (**b**), and oxidases, hydrolases, transferases, and esterases (**c**) in the microsomal fraction of healthy, E ADPKD, and ES‐ADPKD kidney tissues, excluding 0 expressions; the lines depict the mean. Differences between the groups were assessed using the Kruskal‐Wallis and Dunn's tests. (*P < 0.05, **P < 0.01, ***P < 0.001, ****P < 0.0001).

To evaluate the impact of ADPKD on renal DME expression, abundance levels were examined in the microsomal fraction across E‐ADPKD, ES‐ADPKD, and healthy controls. Both median and mean expression data for each group of samples are presented in **Table** **1**. **Figure** **1** shows the individual abundance values of cytochrome P450 enzymes (CYP), UDP‐glucuronosyltransferases (UGTs), and non‐CYP non‐UGT targets. Three UGT enzymes, UGT1A6, UGT1A9, and UGT2B7, were measured in all healthy and E‐ADPKD samples with no significant differences in their levels (*P* > 0.05). However, the ES‐ADPKD samples showed highly variable amounts of these enzymes (**Table** **1**), with the upper end of the range falling within the “healthy” range and the lower below the limit of quantification. The kidney is known to express small amounts of CYP enzymes. We quantified CYP2B6, CYP3A5, and CYP4F2 in different kidney microsomes in a previous targeted analysis.^8^

**Table 1:** Protein expression levels of drug‐metabolizing enzymes in the microsomal fractions of healthy controls, E‐ADPKD, and ES‐ADPKD samples

Protein	Healthy controls					E‐ADPKD					ES‐ADPKD				
	Median^a^	Mean ± SD^a^	Covariance^b^	Range^a^	Count/ 11^c^	Median^a^	Mean ± SD^a^	Covariance^b^	Range^a^	Count/ 16^c^	Median^a^	Mean ± SD^a^	Covariance^b^	Range^a^	Count/ 14^c^
CYP1B1	0	0.05 ± 0.17	316	–	1	0	0.05 ± 0.18	387	–	1	0.11	0.31 ± 0.41	134	0.21–1.14	7
CYP4F2	2.45	2.94 ± 2.58	88	1.4–9.71	9	2.44	2.24 ± 1.73	77	0.97–5.15	12	0	0.09 ± 0.32	361	1.25–1.25	1
UGT1A6	14.88	19.93 ± 16.22	81	5.49–63.19	11	12.08	13.75 ± 6.79	49	5.06–27.94	16	0.4	1.2 ± 1.97	164	0.2–6.82	9
UGT1A9	85.65	91.09 ± 50.34	55	22.69–201.19	11	58.69	56.42 ± 20.91	37	25.13–94.29	16	0.12	8.63 ± 20.54	238	0.24–73.51	7
UGT2B7	127.14	124.54 ± 68.55	55	26.73–222.54	11	82.9	83.03 ± 32.4	39	33.44–147.99	16	0.46	9.54 ± 20.22	212	0.18–58.65	10
FMO1	42.99	51.11 ± 24.18	47	25.01–99.15	11	28.24	31.78 ± 12.06	38	13.44–57.48	16	0	2.95 ± 7.78	264	0.59–29.98	4
FMO3	1.22	2.46 ± 2.85	116	0.4–10.63	10	2.01	2.16 ± 1.74	81	1.03–7.77	14	1.06	1.18 ± 0.52	44	0.38–1.87	14
FMO4	0.38	0.62 ± 0.76	123	0.38–2.38	6	0.27	0.78 ± 1.05	135	0.54–3.64	8	0	0.07 ± 0.23	361	0.91–0.91	1
FMO5	2.08	1.83 ± 0.76	42	0.33–2.77	11	1.27	1.52 ± 1.07	70	0.26–4.07	16	0	0.09 ± 0.27	301	0.01–1.05	3
SULT1C2	4.03	4.19 ± 2.31	55	0.52–8.96	11	4.45	4.92 ± 1.62	33	2.83–8.86	16	0	0.24 ± 0.63	260	0.34–2.43	3
AO	6.03	5.83 ± 3.26	56	0.56–11.97	11	3.73	4.56 ± 2.3	50	1.59–9.83	16	0.06	0.54 ± 1.11	208	0.11–4.23	7
EPHX1	60.14	60.7 ± 24.37	40	23.76–100.65	11	35.01	43.31 ± 19.41	45	25.26–81.38	16	4.93	7.29 ± 7.36	101	1.09–30.82	14
EPHX2	8.3	8.22 ± 2.83	34	2.46–13.69	11	4.3	4.5 ± 1.37	30	2.4–7.12	16	0.28	0.49 ± 0.84	171	0.18–3.14	9
TPMT	1.93	1.94 ± 1.13	58	0.28–3.36	10	1.2	1.06 ± 0.85	80	0.67–2.79	11	0	0.1 ± 0.29	307	0.2–1.13	2
CES 1	0.76	0.72 ± 0.66	92	0.61–2	7	0.56	0.69 ± 0.98	142	0.17–4.19	11	0.4	0.39 ± 0.27	71	0.23–0.91	11
CES 2	16.52	16.02 ± 6	37	5.33–25.89	11	10.63	10.65 ± 4.94	46	3.13–20.4	16	0	1.19 ± 4.15	350	0.1–16.15	3

^a^ pmol/mg of microsomal protein.

^b^ %.

^c^ Number of samples in which the protein could be quantified.

In the present set of samples, CYP4F2 and CYP1B1 were detected in few samples. CYP4F2 showed no significant difference between healthy and E‐ADPKD samples but could only be measured in one ES‐ADPKD sample. CYP1B1 was most abundant in the ES‐ADPKD samples, quantified in 7 out of 14 samples. In the E‐ADPKD samples and healthy controls, CYP1B1 was detected in only one sample. The flavin‐containing monooxygenases (FMOs) 1,3,4 and 5 exhibited a similar pattern in healthy and E‐ADPKD kidneys, with statistically indistinguishable amounts of FMOs. FMO1 and FMO5 were present in all E‐ADPKD and healthy samples but were detected in a few ES‐ADPKD samples (*n* = 4 and *n* = 3 out of 14 samples, respectively). FMO3 was the one enzyme in this group quantifiable in all ESADPKD samples. The median abundances of FMO3 were 1.22, 2.01, and 1.06‐ pmol/mg microsomal protein in healthy, E‐ADPKD, and ES‐ADPKD kidneys, respectively. There were no significant differences in FMO3 abundances between the healthy and diseased samples. Covariance of the FMOs was high for all 3 groups, with the highest variances observed in the ES‐ADPKD kidneys. Sulfotransferase 1C2 (SULT1C2) was quantified in 3 ES‐ADPKD samples out of 14, leading to significantly decreased expression compared with healthy controls and E‐ADPKD samples (*P* < 0.001 and *P <* 0.0001). SULT1C2 showed no significant difference between healthy and E‐ADPKD samples (*P* > 0.05). The 2 epoxide hydrolases showed more than 90% reduced expression in the ES‐ADPKD samples compared to the healthy controls (*P* < 0.0001). EPHX1 and EPHX2 were also significantly lower in the ES‐ADPKD samples than in E‐ADPKD (*P* < 0.0001 and *P* < 0.001). Significant abundance decreases in ES‐ADPKD samples compared to healthy controls and E‐ADPKD were also observed for thiopurine methyltransferase (TPMT) and cocaine esterase (CES2).

Aldehyde oxidase (AO) is a cytosolic enzyme but is often found in microsomes.^34^ It was detected (median: 6.03 pmol/mg microsomal protein and 3.73 pmol/mg microsomal protein) in all healthy E‐ADPKD samples and in 7 out of the 14 ES‐ADPKD samples (median: 0.06 pmol/mg, limit of quantification abundance: 0.01 pmol/mg protein). This decrease was statistically significant compared to healthy controls and E‐ADPKD samples (100‐fold, *P* < 0.0001 and 62.2‐fold, *P* < 0.001, respectively). Liver esterase (CES1) differed from other DMEs, as no statistically significant difference was observed between the diseased and healthy samples (*P* > 0.05). **Table** **1** shows that the median abundances of all reported enzymes, except CYP1B1, FMO3, and SULT1C2, were decreased in the E‐ADPKD samples compared to the healthy control. However, none of the changes were statistically significant. Notably, EPHX2 showed a 1.93‐fold decrease and was narrowly non‐significant (*P =* 0.08).

#### Drug transporters

**Figure 2:**
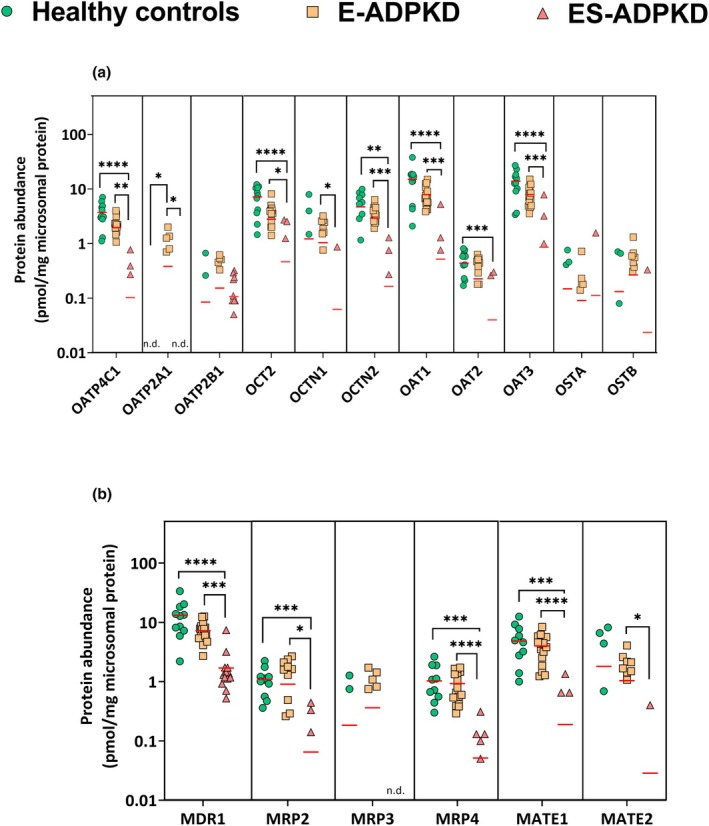
Total abundance of uptake transporters (**a**) and efflux transporters (**b**) in the microsomal fraction of healthy, E‐ADPKD, and ES‐ADPKD kidney tissues, excluding 0 expressions; the lines depict the mean. Differences between the groups were assessed using the Kruskal‐Wallis and Dunn's test. (*P < 0.05, **P < 0.01, ***P < 0.001, ****P < 0.0001).

**Table** **2** shows the quantification of 6 efflux transporters (4 ABC transporters: MDR1 and MRP2‐4 and 2 solute carriers: MATE1 and MATE2) and 11 uptake transporters. Very few transporters could be quantified in all or most of the samples. OAT1, OAT3, and MDR1 were quantified in all the healthy and E‐ADPKD samples but in only 3, 3, and 13 ES‐ADPKD samples (out of 14), respectively. **Figure** **2** and **Table** **2** show considerable overlap between transporter expression in the healthy and E‐ADPKD kidneys but much reduced expression in the ES‐ADPKD kidneys.

**Table 2:** Protein expression levels of drug transporters in the microsomal fractions of healthy controls, E‐ADPKD, and ES‐ADPKD samples

Protein	Healthy controls					E‐ADPKD					ES‐ADPKD				
	Median^a^	Mean ± SD^a^	Covariance^b^	Range^a^	Count/ 11^c^	Median^a^	Mean ± SD^a^	Covariance^b^	Range^a^	Count/ 16^c^	Median^a^	Mean ± SD^a^	Covariance^b^	Range^a^	Count/ 14^c^
OATP4C1	3.19	3.73 ± 1.73	46	1.11–7.06	11	2.03	1.97 ± 1.04	53	1.06–3.99	14	0	0.1 ± 0.22	214	0.27–0.77	3
OATP2A1	0	‐	‐	‐	0	0	0.38 ± 0.62	163	0.7–2	5	0	‐	‐	‐	0
OATP2B1	0	0.08 ± 0.2	236	0.26–0.67	2	0	0.15 ± 0.23	152	0.33–0.62	5	0.09	0.11 ± 0.11	103	0.05–0.32	9
OCT2	7.55	7.14 ± 3.59	50	1.46–12.08	11	3.07	2.79 ± 2.18	78	1.41–8.11	12	0	0.46 ± 0.94	202	1.24–2.7	3
OCTN1	0	1.22 ± 2.43	199	1.48–7.95	3	0.38	1.04 ± 1.16	112	0.76–3.2	8	0	0.06 ± 0.22	361	0.87–0.87	1
OCTN2	5.29	4.7 ± 3.3	70	1.16–9.95	9	2.97	2.93 ± 1.78	61	1.9–6.38	13	0	0.16 ± 0.37	224	0.27–1.28	3
OAT1	16.12	14.97 ± 9.28	62	2.08–37.78	11	7.13	7.92 ± 3.25	41	3.85–14.97	16	0	0.52 ± 1.35	261	0.76–5.21	3
OAT2	0.41	0.44 ± 0.25	57	0.17–0.81	10	0.18	0.23 ± 0.23	101	0.18–0.63	9	0	0.04 ± 0.1	246	0.26–0.3	2
OAT3	12.92	13.85 ± 7	51	3.37–26.95	11	7.18	7.87 ± 3.13	40	3.54–15.07	16	0	0.86 ± 2.1	245	1–7.82	3
OSTA	0	0.15 ± 0.26	172	0.41–0.76	3	0	0.09 ± 0.18	200	0.14–0.72	5	0	0.11 ± 0.4	361	1.57–1.57	1
OSTB	0	0.13 ± 0.26	199	0.08–0.71	3	0	0.27 ± 0.36	137	0.31–1.31	7	0	0.02 ± 0.08	361	0.33–0.33	1
MDR1	12.79	13.09 ± 8.24	63	2.2–33.57	11	6.89	7.22 ± 2.62	36	2.71–12.57	16	1.24	1.69 ± 1.72	102	0.52–7.37	13
MRP2	1.07	1.08 ± 0.52	48	0.36–2.23	11	0.39	0.91 ± 0.95	105	0.26–2.67	10	0	0.07 ± 0.14	211	0.14–0.44	3
MRP3	0	0.18 ± 0.41	221	0.75–1.27	2	0	0.36 ± 0.58	159	0.75–1.72	5	0	‐	‐	‐	0
MRP4	0.72	1.03 ± 0.77	75	0.3–2.63	10	0.72	0.93 ± 0.5	54	0.29–1.73	16	0	0.05 ± 0.09	169	0.05–0.31	5
MATE1	4.6	4.84 ± 3.62	75	1–12.51	10	4.07	3.99 ± 1.97	49	1.23–8.36	16	0	0.19 ± 0.39	207	0.65–1.34	3
MATE2	0	1.81 ± 2.93	162	0.69–8.18	4	0.54	1.04 ± 1.21	117	1.07–4.07	8	0	0.03 ± 0.1	361	0.4–0.4	1

^a^ pmol/mg of microsomal protein.

^b^ %.

^c^ Number of samples in which the protein could be quantified.

In healthy control and E‐ADPKD samples, OAT1, OAT3, and MDR1 showed the highest expression levels. In the ES‐ADPKD samples, MDR1 was the only efflux transporter whose median concentration was above the limit of quantification (1.24 pmol/mg microsomal protein, *n* = 13). In the E‐ADPKD group, OAT1, OAT3, and MDR1 were decreased relative to healthy controls, with reductions of 2.26, 1.80, and 1.86‐fold, respectively. However, none of these changes were statistically significant. All 3 proteins were significantly decreased in the ES‐ADPKD samples compared to both the healthy controls (*P* < 0.0001) and EADPKD samples (*P* < 0.001). MDR1 expression was 10.3‐fold and 5.56‐fold lower than in healthy controls and E‐ADPKD samples. The fold decrease for OAT1 and OAT3 could not be accurately determined, as the median concentration in ES‐ADPKD was below the limit of quantification. However, assuming a lower limit of quantification of 0.01 pmol/mg microsomal protein, the reduction was estimated to exceed 1200fold.

The uptake transporter OAT2 and OATP4C1 were 2.27‐ and 1.57‐fold reduced in the E‐ADPKD samples, but these changes were not statistically significant (*P =* 0.14 and *P =* 0.16). OCT2 showed a 2.45‐fold decrease, which was nearly statistically significant (*P* = 0.05). In the ES‐ADPKD samples, the median expression levels of all 3 transporters were below the limit of quantification. Assuming a lower limit of quantification of 0.01 pmol/mg microsomal protein, the fold changes exceeded 400fold. OCTN2, MRP2, MRP4, and MATE1 did not differ significantly between E‐ADPKD and healthy samples (*P* > 0.05), but in the ESADPKD‐ group, all 4 transporters were significantly lower compared to healthy controls (*P* < 0.001). The transporters OCTN1 and MATE2 were only quantified in a few samples in each group, with a median concentration below the limit of quantification. Healthy and diseased samples, even ES‐ADPKD, did not differ significantly. However, significant differences were observed between E‐ADPKD and ES‐ADPKD samples for all 4 transporters.

Similar low abundances were measured for OAT2B1, OSTα, and OST‐β across all samples, with no significant differences between groups. The low abundant OATP2A1 drug transporter was significantly increased in the E‐ADPKD samples compared to both the healthy control and ES‐ADPKD samples (*P* < 0.05). Based on median fold change, eight out of 17 drug transporters were less abundant in the E‐ADPKD samples compared to the healthy controls, though this decrease was never statistically significant.

### Comparison of abundance of drug‐metabolizing enzymes (DMEs) in the cytosolic fraction

**Figure 3:**
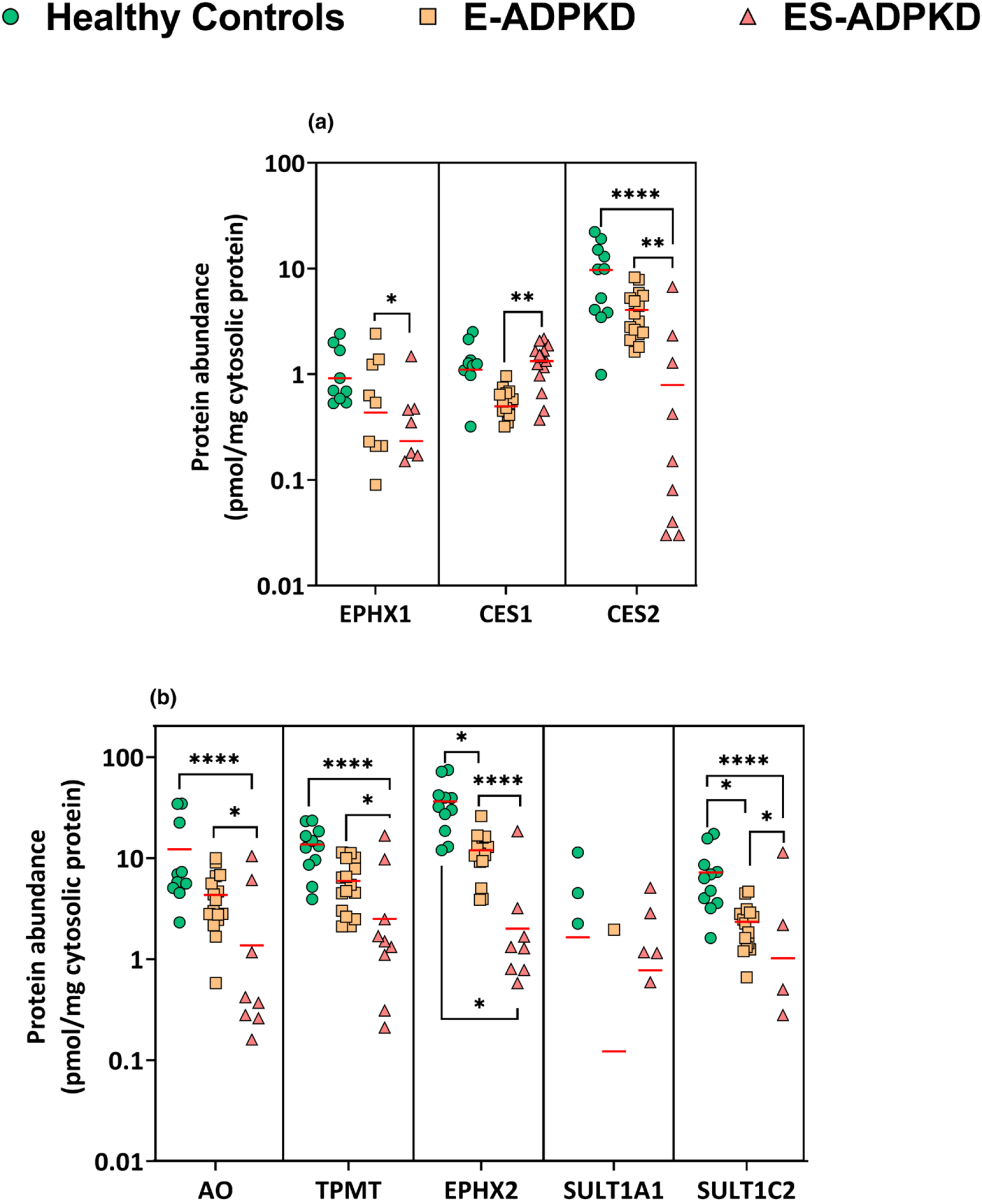
Total abundance of microsomal enzymes (**a**) and cytosolic enzymes (**b**) in the cytosolic fraction of healthy, E‐ADPKD, and ES‐ADPKD kidney tissues, excluding 0 expressions; the lines depict the mean. Differences between the groups were assessed using the Kruskal Wallis and Dunn's test. (*P < 0.05, **P < 0.01, ***P < 0.001, ****P < 0.0001).

Figure 3 shows the abundances of DMEs in the cytosol. CES2, an enzyme typically found in the endoplasmic reticulum, was the most abundant microsomal enzyme detected in the cytosolic fraction. The enzyme was decreased in E‐ADPKD and ES‐ADPKD samples, with reductions of 2.84fold and 245.75fold. The changes in the ES‐ADPKD samples were statistically significant (*P* < 0.0001), while the decrease in the E‐ADPKD samples was not (*P =* 0.07). A previous study in the liver has also quantified CES2 in the cytosolic fraction.^35^

Epoxide hydrolase 1 (EPHX1) levels in ES‐ADPKD samples were significantly lower than in healthy controls, with an 88% decrease (*P* < 0.01). The enzyme CES1 showed a non‐significant increase in ES‐ADPKD samples (1.15fold). EPHX2 and SULT1C2 were 2.7fold and 2.67fold lower in E‐ADPKD samples compared to the healthy controls (*P* < 0.05). Both enzymes, along with AO and TPMT, were decreased by more than 18‐fold in the ES‐ADPKD samples (*P* < 0.0001). The ES‐ADPKD expression levels of all DMEs were significantly lower than those in the E‐ADPKD samples. High variability was again observed in the ES‐ADPKD group, with 6, 5, 6, and 10 samples, respectively, falling below the limit of quantification for EPHX2, TPMT, AO, and SULT1C2 (ES‐ADPKD: *n* = 14). SULT1A1 was low in abundance in all 3 groups and showed no statistical difference between the healthy and diseased samples. A comparison between **Table** **3** and **Table** **1** shows that the microsomal enzymes EPHX1 and CES2 had an overall lower abundance in the cytosolic fractions. In contrast, CES1, an enzyme located in the cytoplasm and endoplasmic reticulum, was more abundant in the cytosolic fraction.

**Table 3:** Protein expression levels of drug‐metabolizing enzymes in the cytosolic fractions of healthy controls, E‐ADPKD, and ES‐ADPKD samples

Protein	Healthy controls					E‐ADPKD					ES‐ADPKD				
	Median^a^	Mean ± SD^a^	Covariance^b^	Range^a^	Count/ 11^c^	Median^a^	Mean ± SD^a^	Covariance^b^	Range^a^	Count/ 16^c^	Median^a^	Mean ± SD^a^	Covariance^b^	Range^a^	Count/ 14^c^
EPHX1	0.69	0.91 ± 0.75	82	0.53–2.41	9	0.15	0.43 ± 0.67	154	0.09–2.42	9	0.08	0.23 ± 0.38	165	0.15–1.48	7
CES 1	1.21	1.1 ± 0.75	68	0.32–2.51	9	0.54	0.5 ± 0.24	49	0.32–0.96	14	1.39	1.33 ± 0.55	41	0.37–2.18	14
CES 2	9.83	9.7 ± 6.64	68	0.99–22.22	11	3.46	4.06 ± 2.01	49	1.63–8.26	16	0.04	0.79 ± 1.76	223	0.03–6.69	9
AO	5.79	12.23 ± 11.64	95	2.31–34.59	11	3.43	4.33 ± 2.57	60	0.58–9.97	16	0.21	1.37 ± 2.96	215	0.16–10.48	8
TPMT	13.21	13.62 ± 6.26	46	3.92–23.53	11	5.09	5.95 ± 3.18	54	2.11–11.38	16	0.71	2.5 ± 4.62	185	0.21–16.67	9
EPHX2	32.26	36.38 ± 19.88	55	11.98–74.39	11	11.96	11.98 ± 5.35	45	3.87–25.97	16	0.68	2 ± 4.64	232	0.58–18.44	8
SULT1A1	0	1.65 ± 3.38	205	2.25–11.41	3	0	0.12 ± 0.47	387	1.96–1.96	1	0	0.78 ± 1.43	185	0.59–5.09	5
SULT1C2	6.32	7.22 ± 4.82	67	1.62–17.4	11	2.37	2.34 ± 1.1	47	0.66–4.66	16	0	1.02 ± 2.92	286	0.28–11.34	4

^a^ pmol/mg of cytosol protein.

^b^ %.

^c^ Number of samples in which the protein could be quantified.

## DISCUSSION

Personalized/precision medicine dosing faces severe challenges in ADPKD. Most patients receive long‐term multidrug treatment. The progressive destruction of normal nephrons by the expansion of renal cysts leads to a continuous decline in glomerular filtration rate (GFR), which makes it difficult to determine appropriate drug doses. Renally excreted drugs are adjusted for the decline in GFR in ADPKD patients.^2^, ^5^, ^15^, ^16^, ^36^, ^37^ Recent research has shown that the decrease of drug‐metabolizing enzymes and transporters (DMET) protein expression in the kidney does not necessarily correlate with GFR decline.^17^, ^19^, ^21^, ^36^, ^38^ Dose adjustments determined purely from GFR are not necessarily the best solution, and quantification of DMET proteins in the ADPKD kidney is paramount. This publication reports the abundances of these proteins for the first time in ADPKD kidneys.

The units used (pmol protein/mg of total protein) align with other studies of this type.^30^, ^39^, ^40^ It is important to note that a decrease in the abundance of a low‐abundance protein can result from two distinct causes. Firstly, the protein of interest may be genuinely less highly expressed. Inflammation and the accumulation of uremic toxins have been shown to lead to the downregulation of DMET proteins.^11^, ^12^, ^13^, ^14^, ^41^, ^42^ However, the upregulation of other proteins, such as immunoglobulins (IGHA1, IGKC, IGHG1) and fibrinogens (FIBA, FIBB) will also lead to reduced DMET expression. The destructive decrease of functional nephron mass is associated with increased interstitial fibrotic tissue, which does not express DMET proteins. The ES‐ADPKD samples were highly fibrotic, which might have hindered the enzymatic protein digestion during sample preparation, resulting in a low amount of digested peptide. However, proteins that are elevated in CKD, such as the fibrinogens (FIBA and FIBB) are increased in the ES‐ADPKD samples compared to the healthy controls.^43^

Yeast alcohol dehydrogenase, used as an internal standard for protein quantification, would remain unaffected, leading to overall decreased protein abundances in the samples if units of absolute abundance were used. The advantage of the unit pmol per mg of total protein is that such potential differences in overall digestion efficiency are accounted for.

Interestingly, several ES‐ADPKD samples showed high expression of DMET protein, reaching levels like those in healthy tissue. This might be due to the samples having more functional tissue and fewer cysts. A lower number of cysts would mean the samples would include less cyst fluid, which contains a high number of proteins.^44^

In the E‐ADPKD samples, we observed a decreased expression of a few specific drug‐metabolizing enzymes (DMEs) and drug transporters, although most of these changes were deemed non‐significant. However, the fold decrease for OCT2 exceeded 2‐fold and was only narrowly non‐significant. The renal expression of transporters is paramount, as the kidney is an important excretory organ. Even marginally decreased levels of drug transporters in E‐ADPKD might affect renal elimination of drugs that are prescribed to ADPKD patients, such as antibiotics, β‐blockers, diuretics, and angiotensin‐II antagonists.^5^, ^6^, ^37^

Previous immunohistological tissue staining has confirmed that cysts express the drug transporters MDR1 and MRP3.^24^, ^27^ However, it is possible that the tubule segment from which a cyst originates may influence which transporters are expressed and their abundance. Furthermore, in the cystic epithelial cells, several membrane proteins, such as the EGF receptor and several Na^+^/K^+^‐ATPase proteins, were mislocalized; this could also apply to drug transporters. These factors could influence how much the xenobiotic reaches the interior of the cysts.^25^, ^27^ In the case of bacterial cyst infections, the abundance of drug transporters on the cyst cells might impact intracystic concentrations.

Changes in enzyme expression in E‐ADPKD kidneys showed that EPHX2 was nearly significantly decreased in the microsomal fraction and significantly decreased in the cytosolic fraction, while sulfotransferase 1C2 (SULT1C2) levels were only reduced in the cytosolic fractions. Although changes in the expression of renal DMEs may impact local drug disposition, the liver remains the main drug eliminating organ. Therefore, to properly assess changes in systemic drug disposition, it is necessary to examine DMET protein expression in the E‐ADPKD and ES‐ADPKD liver.

Current studies use preclinical animal models and biomarker analysis, the results suggesting changes in the expression of specific hepatic transporters in ADPKD.^45^, ^46^ Human ADPKD liver samples are paramount for conclusive statements, and organized collection of ADPKD liver samples is strongly advised.

Our findings in E‐ADPKD kidney provide important insight for ADPKD therapy in which the kidney is the target organ, and renal drug concentrations are highly relevant. A wide array of repurposing candidates, which are metabolized by a broad range of DMEs, is investigated.^47^ In‐depth knowledge of enzyme abundances will prevent the use of contraindicated drugs and doses leading to drug accumulation in the kidney.

ES‐ADPKD kidneys showed an extensive decline in most enzymes. Yet, since renal function has greatly declined, the reduction in ES‐ADPKD renal drug transporter abundance will not factor into systemic drug dispositions. The reduction of most renal enzyme abundances and, thus, renal metabolic capacity in ES‐ADPKD should be considered. Patients with ES‐ADPKD kidneys require dialysis. Currently, drug doses are adjusted to account for the lack of renal excretion but not for the lack of renal drug metabolism.^48^ Dialytic small molecule removal efficiency depends on the molecule's characteristics, such as hydrophilicity and plasma protein binding.^48^ These factors may differ between metabolites and parent drugs. Hence, the severe decrease of DMEs in ES‐ADPKD should be considered in the dosing of certain medications. Our investigations in E‐ADPKD and ES‐ADPKD kidneys have shown renal DMET protein expression to be affected. While this is a significant step toward optimized ADPKD therapy, due to the high tissue heterogeneity of the samples, our analysis does not reflect the total kidney drug‐metabolizing enzyme and transporter capacity. Further analyses that integrate tissue architecture, volumetric analysis, and spatial proteomics will be necessary to estimate whole‐organ abundance better.

By incorporating these and future findings into a physiologically based pharmacokinetic model, we can assess the impact of protein expression on the systemic disposition of drugs relevant for ADPKD therapy, predict drug–drug interactions, and ensure precision dosing.^22^, ^23^

## FUNDING

This project has received funding from the European Union's Horizon 2020 research and innovation programme under the Marie Skłodowska Curie grant agreement No. 955879.

## CONFLICT OF INTEREST

Amin Rostami‐Hodjegan is a part‐time employee of Certara Inc, a provider of biosimulation solutions for pharmaceutical companies. All other authors declared no competing interests for this work.

## AUTHOR CONTRIBUTIONS

A.C.T., D.J.M.P., A.R.‐H., P.W., J.N., J.B., and Z.M.A.‐M. wrote the manuscript. A.C.T., D.J.M.P., P.W., J.N., J.B., and Z.M.A.‐M. designed the research. A.C.T., J.B., and Z.M.A.‐M. performed the research. A.C.T., J.B., and Z.M.A.‐M. analyzed the data. P.W. and J.N. contributed materials/new reagents/analytical tools.

## Supporting information


Data S1.

Data S2.
