# Supplementary material for: Changes in Protein Expression of Renal Drug Transporters and Drug‐Metabolizing Enzymes in Autosomal Dominant Polycystic Kidney Disease Patients
Source: Clin Pharmacol Ther. 2025 May 15;118(3):682–92. doi: 10.1002/cpt.3715 (PMC12355022; doi:10.1002/cpt.3715)
Supplement: Supplementary file 1 — Data S1. [file CPT-118-682-s001.docx]

# **Supplementary Data**

Annika C Tillmann, Dorien J M Peters, Amin Rostami-Hodjegan, Patricia Wilson, Jill Norman, Jill Barber, Zubida M. Al-Majdoub

# **Supplementary Methods**

## **Materials and Methods**

Unless otherwise stated, the chemicals were HLPC grade from Sigma-Alderich (Dorset, UK). The sequencing-grade enzymes Lysyl endopeptidase (Lys-C) and trypsin were purchased from Universal Biologicals Ltd (Cambridge, UK). QconCATs and their non-naturally occurring peptide (NNOP) standards were purchased from Polyquant (Bad Abbach, Germany).

## **Human kidney samples**

The human kidney samples were provided by the PKD Charity-sponsored PKD Biobank at UCL/ Royal Free London NHS Foundation Trust Hospital, London, UK (Collected in US, Ethical agreement number 20772). The samples were collected from Early-stage (E-) ADPKD (n=16, Chronic kidney disease (CKD) stage: 1‑3, age: 19-63), End-stage (ES-) ADPKD patients (n=14, CKD stage: 5, age:31-75 years) as well as the healthy controls (n=11, age: 15-75 years). All tissue samples were collected from Caucasians. Healthy control kidneys were obtained from deceased donors whose kidneys were deemed healthy by both a pathologist and a surgeon. Still, they were considered unsuitable for transplantation due to vascular abnormalities that made surgery too challenging. However, the healthy control tissues showed no abnormalities in renal morphology. Of the control samples, 50% were derived from male patients and 50% from female patients. However, data on the sex of individual donors were not available. The samples were flash-frozen in liquid nitrogen at the source and stored at -70 ˚ C until use. Demographic data of the samples is documented in **Table S1**.

## **Sample preparation**

### **Preparation of kidney fractions**

The kidney samples were lysed using a mechanical homogenizer (Thermo Fisher Scientific, UK) in homogenization buffer pH 7.4 (0.5 mM EDTA, 5 mM histidine, 0.25 M sucrose, 0.5 mM dithiothreitol (DDT) and Pierce protease inhibitor tablet (Thermo Fisher Scientific, UK) at 4-5 ml per gram of renal tissue. The homogenate was centrifuged at 5 000 x g for 20 minutes at 4° C using an Optima L-100 ultracentrifuge (Beckman Coulter, Fullerton, CA). The supernatant was stored in cryovials at -70˚ C, then thawed on ice and centrifuged at 105 000 x g for 75 minutes at 4° C. The supernatant (cytosolic fraction) was pipetted into cryovials. The pellet (microsomal fraction) was resuspended in storage buffer (1.15% KCl, 100 mM Trizma, 0.5 mM EDTA). Both fractions were snap-frozen in liquid nitrogen and stored at -80˚ C.

### **Measurement of total proteins in microsomal and cytosolic fractions**

The Pierce® Microplate BCA Protein Assay Kit (Reducing Agent Compatible) (Thermo Scientific™ Pierce™) was used to determine the total protein content in the microsomal and cytosolic fractions as described in the manufacturer’s manual. The samples were measured in triplicate, and Bovine serum albumin (BSA) was used as a calibration standard. The SpectraMax 190 plate reader (Molecular Devices, Sunnyvale, CA) measured the absorbance at 562 nm.

### **Preparation of kidney fractions for proteomic analysis**

The cytosolic and microsomal fractions were prepared for analysis using filter-aided sample preparation (FASP) as described in previous publications.^1–3^ Each fraction (70 µg) was spiked with yeast alcohol dehydrogenase (YAD, 0.056 µg), bovine serum albumin (BSA, 0.14 µg), TransCAT2 (0.28 µg), MetCAT2 (0.56 µg), and NuncCAT (0.42 µg) as previously described.^4–6^ Sodium deoxycholate (10% w/v final concentration) was added to the samples to solubilize the proteins. Subsequently, 0.1M DDT final concentration in 0.1 M Tris-HCl (pH 8.5) was added to reduce disulfide bonds. After briefly vortexing and centrifuging the samples for 1 minute in a microfuge, they were left at room temperature for 10 minutes. This was followed by 30 minutes of incubation at 56˚ C. Centrifugal filter units (Amicon Ultra, Merck) (MWCO 10 000) were prepared by washing them with 200 µL of 0.1 M Tris-HCl (pH 8.5) and centrifuging them for 10 minutes at 18 410 x g in an Eppendorf® Centrifuge 5424R (Merck). This centrifuge was used in all the following experimental steps. The washing step was performed twice. After adding the samples to the filters, they were centrifuged for 20 minutes at room temperature at 15 870 x g. This was followed by 4 washing steps, which used 200 µL 8 M urea in 0.1 M Tris HCl (pH 8.5) and centrifugation at 18 410 x g for 20 minutes at room temperature. The flow-through was discarded. The reduced cysteines in the samples were alkylated by adding 140 µL of 50 mM iodoacetamide (IAA) and mixing in a thermomixer (600 rpm) at room temperature for 1 minute. The samples were then incubated in the dark for 30 minutes without shaking. After centrifuging the samples for 10 minutes at 18 410 x g at room temperature, they were washed twice using 200 µL 8 M urea and centrifugation at 18 410 x g at room temperature for 20 minutes. Afterward, the samples were washed with 200 µL of 1 M urea in 50 mM ammonium bicarbonate (pH 8) and centrifuged at 15 870 x g at room temperature. This step was conducted twice. The flow-through was discarded after each washing step. The filters were then put into a new collection tube, and 80 µL 1 M urea in 50 mM ammonium bicarbonate (pH 8) was added. This was to prevent evaporation. Lysyl-C Endopeptidase was dissolved in 1 M urea in 50 mM ammonium bicarbonate. The enzyme was added to the sample (enzyme: protein ratio 1:50). After mixing the samples at 600 rpm in the Thermomixer for 1 minute; they were incubated for 3 hours at 30˚ C. Afterwards, Trypsin was reconstituted in 50 mM ammonium bicarbonate solution. The enzyme was added to the samples (enzyme : protein ratio 1:25). After incubating them for 17 hours at 37˚C, the samples were centrifuged at 18410 x g at room temperature for 20 minutes. Subsequently, 100 µL 50 mM NaCl was added to the filters, followed by centrifuging the samples for 20 minutes at 18 410 x g at room temperature. The flow-through volume containing the peptides was measured. Half of the volume was stored at -80˚ C. To the remaining peptides, one-sixth of the initially measured volume of a solution of 2% trifluoracetic acid (TFA) and 20% acetonitrile (ACN) in HLPC water was added. The volume of the flow-through peptides was measured, and one-third of the volume was added to the samples. To clean the samples, they were applied to Pierce^TM^ C‑18 Spin Columns (Thermo Scientific) and the cleaning steps were carried out according to the manufacturer’s instructions. After drying, as per the manual, the samples were stored at -80˚ C.

### **Mass spectrometry analysis**

The samples were reconstituted in 35 µL of loading buffer (3% ACN and 0.1 % formic acid (FA) in HLPC water) containing the unlabelled peptide standards (NNOPs) (**MetCAT2 NNOPs** (0.0672 pmol): GVNDNEEGFFSAR, VGFAFDLPWGSIK, and GISNEGQNASIK, **NuncCAT NNOP** (0.42 pmol): AEGVNDNEEGFFSAR and TEGVNDNEEGFFSAR**, TransCAT2 NNOPs** (0.0224 pmol): VGFLPDGVIK and EGVNDNEEGFFSAR). The samples were loaded on the on the UltiMate 3 000 rapid separation liquid chromatography (RSLC, Dionex, Surrey, UK) using 1 µL per sample (1 µg). A PepMAPRSLC C18 column (Thermo Scientific UK) was used. The buffers A (0.1% FA in HLPC water) and B (0.1% FA in 100% ACN) served as the mobile phase. The run applied a multistep gradient that started with 4% Buffer B and 96% Buffer A. The run ended with 60% Buffer B and 40% Buffer A over 90 minutes. The flow rate of the run was 300 nL/min. The Q Exactive HF Hybrid Quadrupole-Orbitrap mass spectrometer (Thermo Fisher Scientific, Bremen, Germany) was coupled to the LC and collected data for 90 minutes in positive mode from the eluent injected into the LC. The data-dependent analysis selected peptides automatically for fragmentation. The basis for this were the 12 top peptides (m/z 300-1 759 Th, charge 2+ to 4+, dynamic exclusion: 15 seconds). While the MS resolution was set at 120 000 (AGC target 3e6, max. fill time 20 ms), the MS2 resolved at 30 000 (AGC target 2e5, max. fill time 45 ms). In the MS2 resolution, the isolation window was 1.3 Th, and the collision energy was set to 28 eV.

## **Data analysis**

### **Data analysis and protein quantification**

The data was analyzed with MaxQuant version 2.2.3.0 (Max Planck Institute, Martinsried, Germany). A modified human proteome database (UniProtKB proteome UP000005640 fasta file, May 2017), containing 71,790 forward protein sequences, was used for the analysis. The database was modified by adding the protein sequences of the standards (ALBU_BOVIN, ADH1_YEAST, NuncCAT, TransCAT2, and MetCAT2). In the MaxQuant settings, the proteolytic enzymes Trypsin/P and Lys C were chosen, with 1 miscleavage maximally occurring. Methionine oxidation and cysteine carbamidomethylation were selected as variable and fixed modifications, respectively. The settings for mass tolerance were 5 ppm for precursor ions and 0.02 Dalton for fragment ions. Furthermore, the maximal charge was 4+. Peptides shorter than 5 peptides or heavier than 5 000 Daltons were excluded. The peptides taken into account for quantitation were either unmodified or had a methionine oxidation. The subsequent analysis excluded peptides with a score below 40 and reverse peptides.

After running the MaxQuant software, the evidence file was converted into an Excel file for the subsequent data analysis. The evidence file contains peptides and protein identifiers as well as intensities. The protein identifiers were removed and newly assigned. To determine accession, a modified database was built based on a human fasta file (UniProtKB proteome UP000005640 fasta file, May 2017). The data file was called “HUMAN PROTEOME CAPKR 11” and held 1 sequence per protein. The database also listed the priority of individual proteins, with intact and long sequences holding a higher priority than fragments, cDNA-derived peptides, and short sequences. In case no accession could be found for the protein, the full MaxQuant database was used. In that case, these MaxQuant protein accession and their sequences were added to the “HUMAN PROTEOME CAPKR 11”. Peptides that had multiple accessions were deemed non-unique. Accessions for these non-unique peptides were assigned to proteins from which unique peptides had already been detected. This was done for each data set individually.^3^ The quantitation intensities of non-unique peptides were excluded. In the subsequent razor, the priority assigned in “HUMAN CAPKR PROTEOME” and the number of peptides occurring for a protein were used to rank the data. In our analysis, we used a Global Razor. If unique peptides of a specific protein appeared in at least one sample, they were assumed to be present in all other samples. Zero values were explained by the peptides falling under the limit of quantification. Several tutorial videos document the detailed steps in creating a Final Global Razor. Contact the University of Manchester (senior author) to access them.

### **Exclusion of light intensities of QconCATs**

The QconCAT heavy labelling of peptides is assumed to be incomplete. 25 µg QconCATs were digested, reconstituted, run on LC-MS/MS equipment, and analyzed as described above. Individual runs of QconCATs allowed the determination of labelling effectiveness. To account for unlabeled QconCAT, the following steps were taken for each peptide individually.

| **Labelling Efficiency (LE)** | Determines the relative part of the heavy labelled peptides of the total Intensity | $\frac{Heavy Intensity}{Total Intensity}$ | QconCAT analysis |
| --- | --- | --- | --- |
| **Labelling Inefficiency (LIE)** | Determines the relative proportion of light intensity from the unlabeled peptide of the QconCAT | $\frac{Light Intensity}{Total Intensity}$ |  |
| **QconCAT Total Sample Intensity (TSI)** | Determines the Total Intensity (Heavy + Light) of the QconCAT based on the LE | $\frac{Heavy Intensity}{\mathrm{LE}}$ | Sample analysis |
| **QconCAT Light Intensity (QCLI) in Sample** | Determines the light intensity caused by the unlabelled proportion of the labelled QconCAT based on TSI and LIE | TSI · LIE |  |
| **Sample adjustment** | Subtract the light intensity from the unlabelled proportion of the labelled QconCATfrom the total light intensity | Total light intensity - QCLI |  |

The results were further checked using Protein Atlas to confirm that the protein's mRNA is expressed in the kidney. If mRNA expression was not found, the quantified abundance was assumed to originate from the QconCAT peptides, despite prior adjustment. Furthermore, we used Uniport database ([www.uniprot.org/](https://www.uniprot.org/)) to determine the protein’s cellular location and to predict the expected protein fraction.

If a protein was quantified in an unexpected fraction, we investigated whether peptides not stem from QconCAT were detected. If this was the case for more than two fraction samples, the protein was considered to be present in thos fractions. Proteins deemed present were quantified using the intensities of all peptides, and the prior adjustment to account for the unlabeled QconCAT peptides was assumed to be correct.

### **Global analysis**

The final Global Razor was used for quantification using the HiN approach. For each protein, the 3 highest unique peptide intensities were averaged. If only 2 unique peptides had been detected, the 2 highest unique peptide intensities were used instead. Proteins where only 1 unique peptide occurred were assumed to be below the limit of quantification.^5^  The standard YAD, whose initial concentration (21.7 pmol/mg of fractional protein) was known, was then used to calculate the protein concentrations. BSA was excluded as a standard due to highly varying intensities across different samples in the same run.

### **Reproducibility of kidney data**

Technical replicates' similarity was assessed by calculating the Percentage Identical Peptides (PIP) based on the detectability of the peptides. The intersample correlation was evaluated by calculating the PIP and percentage identical Proteins (PIPr). Based on the PIPr values, a principal component analysis (PCA) was performed.^7^

### **Handling of below the limit of quantification (BLQ) concentrations**

The abundances of several proteins investigated in the samples were below the limit of quantification. The limits of quantification for individual proteins and peptides are not known. Therefore, BLQ concentrations could not be assessed for them. The lowest concentration measured varied greatly from sample to sample both in the microsome (Healthy control: 0.02-0.37 pmol/mg microsomal protein, E-ADPKD: 0.05-0.31 pmol/mg microsomal protein, ES-ADPKD: 0.01-0.12 pmol/mg microsomal protein) and cytosol (Healthy control: 0.01 - 0.37 pmol/mg cytosolic protein, E‑ADPKD: 0.03 - 0.19 pmol/mg cytosolic protein, ES-ADPKD: 0.01 - 0.18 pmol/mg cytosolic protein)

The lowest measured protein abundance, across all 3 groups, was 0.01 pmol/mg protein and was simultaneously the lowest limit of quantification (LLOQ). For statistical analysis and fold-change calculations BLQ concentrations were assumed to be 0.01 pmol/mg protein.

### **Statistical Data analysis**

Statistical analysis was conducted with GraphPad Prism 9.3.1 (La Jolla, CA). The data was assessed for normality using the Anderson-Darling, D’Agostino−Pearson, Shapiro−Wilk, and Kolmogorov−Smirnov tests in GraphPad. To determine statistically significant differences between groups (Healthy controls vs. E-ADPKD group, Healthy controls vs. E-ADPKD group, E-ADPKD group vs. E-ADPKD group), the non-parametric Kruskal Wallis test and a post-hoc Dunn’s test, that accounted for the multiple comparisons between the groups, were used.^8^ Changes were deemed statistically significant if the adjusted *P*-value was below 0.05. Importantly, for the statistical analysis, concentrations below the limit of quantification were assumed to be 0.01 pmol/mg of protein, the lowest measurable concentration. In the graphs, concentrations below the limit of quantification assumed to be 0 and were not included. Excel was used to calculate and tabulate the median, mean ± standard deviation, covariance, range, and the number of samples in which the protein abundance was above the limit of quantification. For these calculations, samples with expression levels below the limit of quantification were assumed to have a 0 pmol/mg of protein concentration.

1. **Microsomal and cytosolic protein per gram of kidney (MPPGK and CPPGK)**

MPPGK is usually calculated based on measured abundances in the homogenates of the different samples, while CPPGK has been previously based on the glutathione-S-transferase (GST) activity data found in the cytosolic and microsomal fractions.^9^ Our research analyzed pooled S9 fractions from each group and focused on LC-MS/MS abundance measurements. Furthermore, calculations were performed with estimated microsomal and cytosolic fraction volumes. Calculations of MPPGK and CPPGK were based on the formulas published by Scotcher et al.^9^

We selected CD81, a microsomal marker protein^1,9^, for the MPPGK calculations, as it was quantified in all microsomal samples and remained mostly unquantifiable in the cytosolic fractions. For the CPPGK calculations, we used the abundance levels of GSTK1, a cytosolic glutathione-S-transferase, which was quantified in all cytosolic fractions.^1,9^

### **Medullary and cortical protein markers**

A0AAll tissue samples were sliced in wedges to contain similar amounts of kidney cortex and medulla. Since the medulla does only express low amounts of drug transporters and DMEs, this might impact our measurements. To evaluate whether similar amounts of medulla and cortex are used in the different samples

Aquaporin (AQP) 1 and AQP2 were selected as cortical and medullary markers based on the literature.^10^ The abundances of these proteins were analyzed for each group separately using a GraphPad outlier analysis ([www.graphpad.com/quickcalcs/grubbs1/](https://www.graphpad.com/quickcalcs/grubbs1/)) to investigate potential outliers.

### **Selection of a housekeeping protein for normalization**

The selection of a housekeeping protein required careful evaluation, as ADPKD leads to a wide-ranging dysregulation of cell signaling, cellular growth and energy metabolism.^11,12^ Therefore, statistically significant differences in protein abundance between the 3 groups could result from differences in sample preparation, quality of sample and measurements, or they could also reflect ADPKD-related up- or down-regulation of the protein.

Hence, it is essential to ensure that the selected housekeeping protein is not affected by the disease. The widely used glycolytic enzyme GAPDH (G3P) was excluded, as glycolysis is upregulated in cystic cells in ADPKD.^11,13^ The expression of PARK7, another potential housekeeping protein, is currently being investigated in the context of chronic kidney disease and acute kidney injury.^14,15^ For this reason, PARK7 was also excluded, as its expression may be influenced by the renal manifestations of ADPKD.

Neither cytoplasmic actin 1 (ACTB) nor tubulin β-chain (TBB5) could be quantified across all samples. Thus, cytoplasmic actin 2 (ACTG), which was consistently quantified, was selected as the housekeeping protein. However, it should be noted that it has not been confirmed whether ACTG expression is unaffected by ADPKD, particularly as Polycystin-1 is known to regulate the cytoskeleton organization.^16^

### **Normalization of protein abundances**

HiN intensities of the proteins were normalized by dividing the yeast alcohol dehydrogenase (YAD) standard HiN intensity found in each sample through the ACTG intensity of each sample and subsequently multiplying it by the median HiN intensity of the ACTG protein measured in the healthy controls of the respective samples.

### **Relative distribution of enzymes and transporters**

The DMEs were grouped according to the fraction in which they were quantified, irrespective of their localization. While the drug transporters were split into uptake and efflux drug transporters. Only drug transporters and DMEs previously discussed in this publication were included in the 2 groups. The median concentrations were totaled for each group separately. Proteins with a median value below the limit of quantification were excluded from the percentage calculation.

1. **The 10 most abundant proteins in the microsomal and cytosolic fractions**

The median was used to rank the proteins from highest to lowest abundance in the 3 groups and for both fractions.

# **Supplementary Results**

## **Intensity**

The total proteome MS signal intensities were measured for each sample as a control check. The average total proteome intensity (mean ± standard deviation) in the microsome was 1.43E+12 ± 7.796E+11 and while the mean in the cytosol was 3.51E+12 ± 1.5195E+12 (**Microsome Range: Healthy:** 2.75E+11–3.49E+12, **E-ADPKD:** 8.79E+11–1.32E+12, **E‑ADPKD:**7.31E+11-3.05E+12; **Cytosol Range: Healthy:** 2.36E+12-9.09E+12, **E-ADPKD:** 2.39E+12 – 4.20E+12, **E-ADPKD**: 2.56E+11-4.39E+12). The total proteome intensities of the individual samples are listed in **Table S2**.

## **Percentage identical peptides (PIP) and percentage identical proteins (PIPr) across technical replicates**

The percentage identical peptides (PIP) and percentage identical proteins (PIPr) values of the technical replicates (n=20) from cytosolic and microsomal fractions are listed in **Table S3**. The PIP values of the microsomal technical replicates ranged between 65% and 70% for all 3 groups (**Healthy controls**: 67‑69%, **E-ADPKD:** 65-70%, and **ES-ADPKD:** 63-68%). The lowest PIP value in the microsomal samples was found in the ES-ADPKD samples; however, it was not considered an outlier (63%). The microsomal PIPr values ranged from 77% to 83% (**Healthy controls**: 81-83%, **E-ADPKD:** 81-83%, and **ES-ADPKD:** 77-82%). For the cytosolic samples, the PIP values of technical replicates of E-ADPKD and healthy controls ranged between 64% and 68%, similar to the microsomal technical replicates (**Healthy controls**: 65‑67% and **E-ADPKD:** 64-68%). The cytosolic technical replicates of the ES-ADPKD samples had noticeably lower PIP values than the other 2 groups (**ES-ADPKD:** 51-59%). A previous publication on microsomal kidney samples found PIP values between 63% and 73% for analytical replicates, which aligns with our measurements in this study.^17^ The PIPr values of the cytosolic fractions for the healthy and E-ADPKD samples ranged from 83% to 85% (**Healthy controls**: 83-85% and **E‑ADPKD:** 83-84%), while the ES-ADPKD PIPr values for 2 of the replicates were noticeably lower (**ES-ADPKD:** 74-84%).

## **Percentage identical peptides and percentage identical proteins between samples**

The intersample percentage identical peptides (PIP) and percentage identical proteins (PIPr) values of the technical replicates (n=20) from microsomal and cytosolic fractions are listed in **Table S4** and **Table S5**.

## **Principal component analysis**

In **Figure S1**, the principal component analysis (PCA) is based on the PIPr values between the different samples of the microsomal (**A**) and cytosolic fraction (**B**). PC1 explains a substantial variance (Microsome: 75.8%; Cytosol: 77.01%), while PC2 explains a smaller portion percentage of variance, contributing 5.1% in the microsomal fraction and 6.4% in the cytosolic fraction. In both fractions, E‑ADPKD and healthy controls tended to cluster together, suggesting similarity in the variables measured. The E-ADPKD samples are slightly more spread out, indicating some variability within this group. E-ADPKD samples clustered together regardless of CKD stage, age, and sex of the donor in both fractions. However, cytosolic E-ADPKD samples 12 and 14 (CKD stage 3A) were positioned further from the cluster. The ES‑ADPKD group formed a distinct, more heterogeneous spread rather than a cohesive cluster in both fractions. An exception was ES-ADPKD sample 5, which appeared within the E‑ADPKD/healthy control cluster in the PCA of both fractions. This sample showed the highest abundance of DMET proteins, reaching ranges similar to healthy controls.

In the PCA of the microsomal fraction (**Figure S1A**), the ES‑ADPKD samples 2 and 7 were the second closest to the E-ADPKD/ healthy sample cluster. These samples displayed higher abundances of DMET proteins than other ES-ADPKD samples in the microsomal fraction. In the cytosolic PCA (**Figure S1B**), ES-ADPKD samples 2,6, and 3 clustered close to the healthy/E-ADPKD samples. The ES-ADPKD samples 2, 5, and 7 stem from male donors, while samples 3 and 6 were donated by women. While most ES-ADPKD samples close to the healthy/E-ADPKD cluster were donated by persons in their thirties or forties, the sample within the cluster (sample 5) was collected from a 75-year-old donor. The ES-ADPKD sample position was therefore concluded to be independent from the sex and age of th donor. Furthermore, the ES-ADPKD sample position in the PCA was independent of the total proteome intensity (**Table S2**).

## **Microsomal and cytosolic protein per gram of kidney (MPPGK and CPPGK)**

Based on the abundances of CD81 and GSTK1, we calculated an average MPPGK and CPPGK for each group. The MPPGK values overlapped across the different groups (Healthy Control: 15.2 mg/g kidney; E-ADPKD: 21.6 mg/g kidney; ES-ADPKD: 11.4 ± 8.2 mg/g kidney). Scotcher et al. previously reported an average MPPGK of 26.2 mg protein/g kidney cortex.^9^ While the similarity between our values, especially for healthy controls and E-ADPKD, is noteworthy, direct comparability is limited due to differences in methodology since our values are based on S9 fraction-based estimates. Furthermore, our samples contain a mixture of cortex and medulla. ES-ADPKD showed the lowest MPPGK value.

The average CPPGK values calculated for the healthy control (30.1 mg protein/g kidney) and E-ADPKD (31.0 mg protein/g kidney) were highly similar. Although, both were noticeably lower than the previously reported average of 79.5 mg protein/g kidney cortex. A noticeably, higher CPPGK average was calculated for the ES-ADPKD samples (192.4 mg protein/g kidney).^9^ A possible explanation for this high value is that the cytosolic fractions of the ES-ADPKD samples also contained proteins derived from the cyst fluid. Furthermore, several ES-ADPKD samples contained high GSTK1 abundances, that were not adequately represented in the S9 pool.

## **Selection of the housekeeping protein**

The abundances of the housekeeping proteins G3P and PARK7, which were excluded as their expression might be affected in ADPKD, as well as those of the selected protein ACTG, are displayed in **Figure S2**. Interestingly, abundances of the proteins differed significantly between the 3 groups in both the cytosolic and microsomal fraction (*P* < 0.05). Only the cytosolic abundances of ACTG in the healthy control and ES-ADPKD samples did not significantly differ (p=0.05).

## **Normalized protein abundances**

While the normalization of protein intensities and, subsequently, protein abundances elevated measurements for several samples in the ES-ADPKD groups, it also led to unrealistically high protein levels in healthy controls and drastically decreased abundances in other ES-ADPKD samples.

The comparison of normalized protein abundances impacted the statistical significance of differences for a few specific enzymes and drug transporters. For example, the non-normalized cytosolic abundance of EPHX2 and SULT1C2 in E-ADPKD differed significantly from healthy controls, while the normalized values were no longer significant (*P =* 0.07 and *P =* 0.10, respectively). Similarly, comparisons of normalized microsomal FMO4 and cytosolic CES1 between E- and ES-ADPKD samples were no longer deemed statistically significant (*P =* 0.09 and *P =* 0.08). Additionally, the differences in microsomal OCT2 and MRP2 levels between E- and ES-ADPKD samples also lost statistical significance (*P =* 0.30 and *P =* 0.06). Conversely, the normalized abundances of OATP2B1 and OCTN1 were significantly decreased in ES-ADPKD samples compared to E-ADPKD (*P* < 0.05). Despite these changes, all normalized protein abundances in ES-ADPKD DMET remained statistically significantly different from the healthy controls. The normalized abundances of enzymes and transporters are shown in **Figure S3-S5**.

## **Assessment of cortical and medullary markers in healthy control, E-ADPKD and ES-ADPKD samples**

Previous LC-MS/MS proteomics used the microsomal protein aquaporin-1 (AQP1) and aquaporin-2 (AQP2) as cortical and medullary markers, respectively, to investigate contamination of kidney cortex samples with renal medulla.^10^ AQP1 was detectable in most microsomal samples (n=11 out of 11; E-ADPKD: n=16 out of 16; ES-ADPKD: n=8 out of 14). AQP1 was highly abundant in the healthy control group (median: 76.3 pmol/mg microsomal protein). In E- and ES-ADPD samples, AQP1 levels were significantly decreased compared to the healthy controls (26.2 pmol/mg microsomal protein and 0.59 pmol/mg microsomal protein). These differences were statistically significant between all sample groups (*P* < 0.05). However, since ADPKD disrupts multiple cell signaling pathways, the observed decrease in APQ1 expression may not derive from a lower amount of cortex included in the analysis. Instead, the dysregulated cell signaling in ADPKD might lead to a decreased protein expression in the diseased tissue.^18^ Devuyst et al. documented in their study a stepwise decrease in AQP1 expression in E- and ES-ADPKD kidney tissue samples.^19^

Due to the extensive dysregulation in ADPKD, altered protein expression in ADPKD cannot be excluded for any protein. Outlier analysis within individual groups showed no specific outliers in the healthy control and E-ADPKD group. However, due their high abundance of AQP1, ES‑ADPKD samples 5 and 7 were deemed as outliers within the ES-ADPKD group. Interestingly, in the PCA of the microsomal samples (**Figure S1**), sample 5 was found in the E-ADPKD/healthy control cluster, while sample 7 was close to it.

The abundances of the medullary marker AQP2 were not measurable in the healthy control and E-ADPKD samples. The protein was measurable in 4 out of 14 ES-ADPKD samples. This elevation was nearly statistically significant between the healthy control and ES-ADPKD (*P*= 0.055), while it was significant between the E- and ES-ADPKD samples (*P* < 0.05). Interestingly, a previous publications had found that in E- and ES-ADPKD kidneys, the absolute amounts of AQP2 were significantly decreased. In ADPKD, renal cysts exclusively express either AQP1 or AQP2. This exclusive expression pattern is maintained through the progression of the disease, including in the end-stage of the disease.^19^

It can be hypothesized that depending on the cyst’s origin (proximal tubule or distal tubules/collecting duct), either AQP1 or AQP2 are expressed. In ES-ADPKD, most cysts derive from the distal tubules and collecting duct.^20^ In consequence, our elevated AQP2 measurements could reflect either increased amounts of medulla in the samples or be due to distal/collecting duct cysts being included in the sample.

## **Assessment of markers in microsomal and cytosolic kidney fractions**

For quality control, calnexin (CANX) (ER-marker) and CD81 antigen (CD81) (plasma membrane marker) were selected as microsomal markers.^1^ Both markers were consistently detected in the microsomal fractions of healthy controls, E-ADPKD and ES-ADPKD samples (**CALX:** 50.7 pmol/mg microsomal protein, 41.3 pmol/mg microsomal protein and 9.58 pmol/mg microsomal protein; **CD81:** 23.8 pmol/mg microsomal protein, 21.2 pmol/mg microsomal protein and 30.0 pmol/mg microsomal protein). Notably, in the ES-ADPKD samples, CALX levels decreased more than 5-fold, while CD81 levels increased more than 1.2-fold. In the cytosolic fractions, the median concentrations of CD81 were below the quantification limit in all 3 groups, with CD81 only detected in 2 E-ADPKD and 3 healthy controls. Low concentrations of CANX were quantified in the cytosolic fractions of all 3 sample groups (**Healthy controls:** 1.89 pmol/cytosolic protein, n=7; **E-ADPKD:** 0.72 pmol/cytosolic protein, n=11; **ES-ADPKD:** 0.56 pmol/cytosolic protein, n=12). L-lactate dehydrogenase B chain (LDHB), a cytosolic marker was quantifiable in both the cytosolic fraction (Median: **Healthy controls:** 313.81 pmol/cytosolic protein, n=11; **E-ADPKD:** 191.61 pmol/cytosolic protein, n=16; **ES-ADPKD:** 38.66 pmol/cytosolic protein, n=14) and microsomal fraction (Median **Healthy controls:** 165.88 pmol/microsomal protein, n=11; **E-ADPKD:** 81.79 pmol/microsomal protein, n=16; **ES-ADPKD:** 5.58 pmol/microsomal protein, n=14).

## **The ten most abundant proteins in the microsomal and cytosolic fraction**

The ten most abundant proteins for both fractions in all 3 groups are shown in **Table S6**. Cytoplasmic actin and serum albumin were highly abundant in the cytosolic and microsomal fractions across all 3 groups. In the microsomal fractions of healthy controls and E-ADPKD, other highly abundant proteins included aminopeptidase (AMPN) and Fructose-bisphosphate aldolase B (ALDOB). However, both proteins were significantly reduced in the ES-ADPKD samples. In both the microsomal and cytosolic fraction of the ES-ADPKD samples, 2 immunoglobulins were highly expressed. Both proteins had low abundance in the microsomal and cytosolic fractions of healthy controls and E-ADPKD. Glyceraldehyde-3-phosphate dehydrogenase was highly abundant in the cytosolic fraction of all 3 groups; however, in the ES-ADPKD samples, it ranked only as the 23^rd^ most abundant protein.

## **Relative distribution of enzymes and transporters in microsomal and cytosolic fraction**

### **Microsomal and Cytosolic Drug-Metabolizing Enzymes**

The pie charts in **Figure S6** and **Figure S7** show the proportions of DMEs in the microsomal and cytosolic fractions, respectively, for healthy, E-ADPKD, and ES-ADPKD samples. Apart from EPHX2, TPMT, and CES2, the absolute abundances of DMEs in the microsome were not significantly different from those of healthy controls. Similarly, no major differences in relative abundances in the microsomal fraction were observed between the E-ADPKD and healthy control samples. However, in the ES-ADPKD samples, these enzymes were markedly decreased, with EPHX1 and FMO3 being the most abundant enzymes. EPHX1 was the most abundant enzyme in the cytosolic fractions of healthy controls and E-ADPKD samples, and it was the second most abundant DME in the ES-ADPKD samples.

### **Drug transporters**

**Figure S8A** and **S8B** display the relative proportions of uptake and efflux transporters for healthy controls and E-ADPKD, respectively. It was not possible to calculate relative proportions for the ES-ADPKD samples, as most transporters were below the limit of quantification, resulting in the median also falling below the limit of quantification. The only exceptions were the efflux transporter MDR1 and the uptake transporter OATP2B1.

The most abundant uptake transporters were OAT3, OAT1, and OCT2. Although the absolute expression levels of all 3 transporters were significantly decreased in E-ADPKD, the relative proportions did not change drastically between healthy controls and E-ADPKD.

The most abundant efflux transporters were MDR1 and MATE1. No statistically significant difference in MRP2 protein expression was observed between healthy controls and E-ADPKD. However, in the proportional assessment, MRP2 showed a decreased percentage of E-ADPKD compared to healthy controls, even when accounting for the general reduction in transporter protein levels.

# **Supplementary Discussion**

In our analysis of healthy control and diseased kidney tissue samples, it was important to consider whether each group contained comparable proportions of medulla and cortex. Since drug-metabolizing enzymes and transporters (DMET) are mainly expressed in the proximal tubules located in the cortex, a higher proportion of medulla in diseased sample could potentially skew the results. The tissue samples have been cut to contain approximately equal amounts of cortex and medulla, making a consistent error implausible. To further confirm this, we investigated cortical and medullary marker proteins across the different disease groups. Aquaporin (AQP) 1 and AQP2 are widely recognized cortical and medullary markers, respectively.^10,19^ Tolvaptan, the only officially released treatment for ADPKD, leads to decreased AQP2 levels. However, since all but one sample were collected before tolvaptan was released as ADPKD treatment on the market (in 2015), this was not considered an issue.^21^

AQP1 and AQP2 have previously been investigated in ADPKD immunohistological tissue stainings. It was demonstrated that even in ES-ADPKD, cysts exclusively express either AQP1 or AQP2.^19^

The expression of AQP1 or AQP2 likely depends on whether the cyst arises from the proximal tubule (AQP1) or the distal tubule/collecting duct (AQP2). It was shown that in both E- and ES-ADPKD, AQP1 and AQP2 levels decreased compared to healthy controls.^19^

Our own analysis found a significant decrease in AQP1 in both E- and ES-ADPKD compared to healthy controls. Despite these reductions, we selected AQP1 and AQP2 as cortical and medullary marker proteins, respectively, to perform outlier analysis for each group.

There are several reasons for this. Firstly, due to the extensive dysregulation of cell signaling and energy metabolism in ADPKD, no protein expression can be assumed to remain unaffected by the disease.^11,13,18^ For AQP1 and AQP2, it has been confirmed that no upregulated or mislocalized expression occurs in the cysts that would distort the findings.

We found no specific outliers in AQP1 and AQP2 abundance in the healthy control and E-ADPKD samples. However, in the ES-ADPKD group, there were significant outliers for both AQP1 and AQP2. AQP2 was only measurable in the ES-ADPKD samples, which may simply reflect a higher amount of medulla or increased amount of distal and medullary cysts in those samples.

Potential alterations in protein expression due to ADPKD had to be carefully considered when selecting a housekeeping protein for normalization. In ADPKD, glycolysis is favored as an energy source.^11,13^ As a result, the glycolytic enzyme, Glyceraldehyde-3-phosphate dehydrogenase (G3P), which is often used as a housekeeping protein, was excluded as an option for this analysis. Another commonly used housekeeping protein, PARK7, was also excluded, as it has been implicated in chronic kidney disease and acute kidney injury, and its expression in ADPKD might therefore be affected.^14,15^ Lastly, the cytoskeletal proteins β-actin and β-tubulin were not consistently quantifiable in all samples.

Cytoplasmic actin 2 (ACTG) was selected as a housekeeping protein due to its role as an actin protein and its reliable quantification in all samples. However, it must be noted that ACTG expression may still be influenced by ADPKD, as Polycystin-1 regulates cytoskeletal organization, and fibrosis occurring in ADPKD might also impact its expression.^16,22^ Although, the normalized results in our analysis were generally consistent compared to the non-normalized ones, the potential limitations related to housekeeping protein variability mean that, at this stage, the normalized values should be interpreted with caution.

# **Supplementary References**

1. Couto N, Al-Majdoub ZM, Achour B, Wright PC, Rostami-Hodjegan A, Barber J. Quantification of Proteins Involved in Drug Metabolism and Disposition in the Human Liver Using Label-Free Global Proteomics. *Mol Pharm*. 2019;16(2):632-647. doi:10.1021/acs.molpharmaceut.8b00941

2. Howard M, Achour B, Al-Majdoub Z, Rostami-Hodjegan A, Barber J. GASP and FASP are Complementary for LC–MS/MS Proteomic Analysis of Drug-Metabolizing Enzymes and Transporters in Pig Liver. *Proteomics*. 2018;18(24):1800200. doi:10.1002/PMIC.201800200

3. Al-Majdoub ZM, Achour B, Couto N, et al. Mass spectrometry-based abundance atlas of ABC transporters in human liver, gut, kidney, brain and skin. *FEBS Lett*. 2020;594(23):4134-4150. doi:10.1002/1873-3468.13982

4. Vasilogianni AM, El-Khateeb E, Al-Majdoub ZM, et al. Proteomic quantification of perturbation to pharmacokinetic target proteins in liver disease. *J Proteomics*. 2022;263(104601). doi:10.1016/j.jprot.2022.104601

5. El-Khateeb E, Al-Majdoub ZM, Rostami-Hodjegan A, Barber J, Achour B. Proteomic quantification of changes in abundance of drug-metabolizing enzymes and drug transporters in human liver cirrhosis: Different methods, similar outcomes. *Drug Metabolism and Disposition*. 2021;49(8):610-618. doi:10.1124/dmd.121.000484

6. Vasilogianni AM, El-Khateeb E, Achour B, et al. A family of QconCATs (Quantification conCATemers) for the quantification of human pharmacological target proteins. *J Proteomics*. 2022;261(104572). doi:10.1016/J.JPROT.2022.104572

7. Al Feteisi H, Al-Majdoub ZM, Achour B, Couto N, Rostami-Hodjegan A, Barber J. Identification and quantification of blood–brain barrier transporters in isolated rat brain microvessels. *J Neurochem*. 2018;146(6):670-685. doi:10.1111/JNC.14446

8. Erdmann P, Bruckmueller H, Martin P, et al. Dysregulation of Mucosal Membrane Transporters and Drug-Metabolizing Enzymes in Ulcerative Colitis. *J Pharm Sci*. 2019;108(2):1035-1046. doi:10.1016/j.xphs.2018.09.024

9. Scotcher D, Billington S, Brown J, et al. Microsomal and cytosolic scaling factors in dog and human kidney cortex and application for in vitro-in vivo extrapolation of renal metabolic clearance. *Drug Metabolism and Disposition*. 2017;45(5):556-568. doi:10.1124/DMD.117.075242/-/DC1

10. Li CY, Hosey-Cojocari C, Basit A, Unadkat JD, Leeder JS, Prasad B. Optimized Renal Transporter Quantification by Using Aquaporin 1 and Aquaporin 2 as Anatomical Markers: Application in Characterizing the Ontogeny of Renal Transporters and Its Correlation with Hepatic Transporters in Paired Human Samples. *AAPS Journal*. 2019;21(5). doi:10.1208/s12248-019-0359-1

11. Podrini C, Rowe I, Pagliarini R, et al. Dissection of metabolic reprogramming in polycystic kidney disease reveals coordinated rewiring of bioenergetic pathways. *Commun Biol*. 2018;1(1). doi:10.1038/s42003-018-0200-x

12. Harris PC, Torres VE. Genetic mechanisms and signaling pathways in autosomal dominant polycystic kidney disease. *Journal of Clinical Investigation*. 2014;124(6):2315-2324. doi:10.1172/JCI72272

13. Podrini C, Cassina L, Boletta A. Metabolic reprogramming and the role of mitochondria in polycystic kidney disease. *Cell Signal*. 2020;67. doi:10.1016/j.cellsig.2019.109495

14. Wiśniewski JR, Mann M. A proteomics approach to the protein normalization problem: Selection of unvarying proteins for MS-based proteomics and western blotting. *J Proteome Res*. 2016;15(7):2321-2326. doi:10.1021/acs.jproteome.6b00403

15. Yin L, Li H, Liu Z, et al. PARK7 Protects Against Chronic Kidney Injury and Renal Fibrosis by Inducing SOD2 to Reduce Oxidative Stress. *Front Immunol*. 2021;12. doi:10.3389/fimmu.2021.690697

16. Yao G, Su X, Nguyen V, et al. Polycystin-1 regulates actin cytoskeleton organization and directional cell migration through a novel PC1-pacsin 2-N-wasp complex. *Hum Mol Genet*. 2014;23(10):2769-2779. doi:10.1093/hmg/ddt672

17. Al-Majdoub ZM, Scotcher D, Achour B, Barber J, Galetin A, Rostami-Hodjegan A. Quantitative Proteomic Map of Enzymes and Transporters in the Human Kidney: Stepping Closer to Mechanistic Kidney Models to Define Local Kinetics. *Clin Pharmacol Ther*. 2021;110(5):1389-1400. doi:10.1002/CPT.2396

18. Harris PC, Torres VE. Genetic mechanisms and signaling pathways in autosomal dominant polycystic kidney disease. *Journal of Clinical Investigation*. 2014;124(6):2315-2324. doi:10.1172/JCI72272

19. Devuyst Oliver, Burrow Christopher R, Smith Barbara L, Agre Peter, Knepper Mark A, Wilson Patricia D. Expression of aquaporins-1 and -2 during nephrogenesis and in autosomal dominant polycystic kidney disease. *Am J Physiol* . 1996;271(1 Pt 2):F169-F183.

20. Terryn S, Ho A, Beauwens R, Devuyst O. Fluid transport and cystogenesis in autosomal dominant polycystic kidney disease. *Biochim Biophys Acta Mol Basis Dis*. 2011;1812(10):1314-1321. doi:10.1016/j.bbadis.2011.01.011

21. Sans-Atxer L, Joly D. Tolvaptan in the treatment of autosomal dominant polycystic kidney disease: Patient selection and special considerations. *Int J Nephrol Renovasc Dis*. 2018;31(11):41-51. doi:10.2147/IJNRD.S125942

22. Parrish AR. The cytoskeleton as a novel target for treatment of renal fibrosis. *Pharmacol Ther*. 2016;166:1-8. doi:10.1016/j.pharmthera.2016.06.006

# **Web references**

[www.uniprot.org/](http://www.uniprot.org/) (06/11/2024)

[www.nhs.uk](http://www.nhs.uk) (06/11/2024)

[www.proteinatlas.org](http://www.proteinatlas.org) (06/11/2024)

[www.graphpad.com/quickcalcs/grubbs1/](http://www.graphpad.com/quickcalcs/grubbs1/) (13/02/2025)
