# Supplementary material for: Changes in Protein Expression of Renal Drug Transporters and Drug‐Metabolizing Enzymes in Autosomal Dominant Polycystic Kidney Disease Patients
Source: Clin Pharmacol Ther. 2025 May 15;118(3):682–92. doi: 10.1002/cpt.3715 (PMC12355022; doi:10.1002/cpt.3715)
Supplement: Supplementary file 2 — Data S2. [file CPT-118-682-s002.docx]

**Table S1.** Demographic information on healthy control (HC), Early-stage (E-ADPKD) and End-stage (ES-ADPKD) kidneys. All tissue samples stemmed from Caucasian donors. (- = no further information)

| **Healthy controls** | | | | | | |
| --- | --- | --- | --- | --- | --- | --- |
| **Sample name** | **Age**  **(years)** | **Sex** | **PKD mutation** | **CKD stage ^a^** | **Tissue sample weight**  **(g)** | **Further information** |
| HC_1 | 15 | Unknown | no mutation | 0 | 0.23 | - |
| HC_2 | 18 | Female | no mutation | 0 | 0.25 | - |
| HC_3 | 20 | Female | no mutation | 0 | 0.37 | - |
| HC_4 | 27 | Unknown | no mutation | 0 | 0.67 | - |
| HC_5 | 31 | Unknown | no mutation | 0 | 0.44 | - |
| HC_6 | 35 | Unknown | no mutation | 0 | 0.89 | - |
| HC_7 | 39 | Unknown | no mutation | 0 | 0.53 | - |
| HC_8 | 49 | Unknown | no mutation | 0 | 1.03 | - |
| HC_9 | 58 | Male | no mutation | 0 | 0.36 | - |
| HC_10 | 67 | Unknown | no mutation | 0 | 1.03 | - |
| HC_11 | 75 | Unknown | no mutation | 0 | 0.42 | - |
| Only limited information is available on the sex of individual healthy control tissue donors. However, it is known that tissue derived from male and female donors in equal parts (50% male and 50% female). | | | | | | |
| **Early-stage ADPKD** | | | | | | |
| **Sample name** | **Age**  **(years)** | **Sex** | **PKD mutation** | **CKD stage ^a^** | **Tissue sample weight**  **(g)** | **Further information** |
| E_1 | 31 | Female | PKD1 pR3753Q | 3A | 0.81 | Fibrotic |
| E_2 | 63 | Female | Unknown | 3A | 1.05 | - |
| E_3 | 47 | Male | Unknown | 3A | 1.09 | - |
| E_4 | Unknown | Unknown | Unknown | Unknown | 0.75 | - |
| E_5 | 32 | Male | Unknown | 3A | 0.55 | - |
| E_6 | 39 | Female | Unknown | 1 | 0.57 | - |
| E_7 | 28 | Female | Unknown | 3 | 0.76 | - |
| E_8 | 57 | Female | PKD1pA3053T | 2 | 0.46 | Fibrotic |
| E_9 | Unknown | Unknown | Unknown | Unknown | 0.40 | - |
| E_10 | 61 | Female | Unknown | 1 | 0.54 | - |
| E_11 | 19 | Male | Unknown | 3A | 0.35 | - |
| E_12 | 27 | Female | Unknown | 3A | 1.10 | - |
| E_13 | 48 | Male | Unknown | Unknown | 0.83 | - |
| E_14 | 34 | Unknown | Unknown | 3A | 0.88 | - |
| E_15 | 36 | Female | Unknown | 3A | 0.61 | - |
| E_16 | 47 | Female | Unknown | 2 | 0.29 | - |
| **End-stage ADPKD** | | | | | | |
| **Sample name** | **Age**  **(years)** | **Sex** | **PKD mutation** | **CKD stage ^a^** | **Tissue sample weight**  **(g)** | **Further information** |
| ES_1 | 39 | Female | Unknown | 5 | 0.30 | - |
| ES_2 | 46 | Male | Unknown | 5 | 0.43 | - |
| ES_3 | 31 | Female | Unknown | 5 | 0.37 | - |
| ES_4 | 41 | Female | Unknown | 5 | 0.23 | - |
| ES_5 | 75 | Male | Unknown | 5 | 0.70 | - |
| ES_6 | 34 | Female | Unknown | 5 | 0.62 | - |
| ES_7 | 44 | Male | Unknown | 5 | 0.37 | - |
| ES_8 | 72 | Unknown | Unknown | 5 | 0.27 | - |
| ES_9 | 65 | Female | Unknown | 5 | 0.22 | - |
| ES_10 | 56 | Male | Unknown | 5 | 0.19 | - |
| ES_11 | 63 | Unknown | Unknown | 5 | 0.16 | - |
| ES_12 | 55 | Male | Unknown | 5 | 0.43 | - |
| ES_13 | 40 | Male | Unknown | 5 | 0.12 | - |
| ES_14 | 43 | Female | Unknown | 5 | 0.20 | - |

a CKD stage is assessed based on estimated glomerular filtration rate (eGFR)- and/or indication of kidney damage through other tests (www.nhs.uk)

| **CKD stage** | **eGFR** | **Kidney damage** |
| --- | --- | --- |
| 0 | ≥90 ml/min, | No |
| 1 | ≥90 ml/min | Yes |
| 2 | 60-89 ml/min | Yes |
| 3a | 45-59 ml/min | Yes |
| 3b | 30-44ml/min | Yes |
| 4 | 15-29 ml/min | Yes |
| 5 | <15 ml/min | Yes |

**Table S2**. Total Intensity measured per sample. (HC= Healthy Controls, E= Early-Stage ADPKD, ES=End Stage-ADPKD).

|  |  |  |  |  |  |
| --- | --- | --- | --- | --- | --- |
| **Microsomal fraction** | | |  | **Cytosolic fraction** | |
| **Sample** | | **Total Intensity** |  | **Sample** | **Total Intensity** |
| HC_1 | | 1.51E+12 |  | HC_1 | 4.85E+12 |
| HC_2 | | 2.75E+11 |  | HC_2 | 3.19E+12 |
| HC_3 | | 4.68E+11 |  | HC_3 | 3.03E+12 |
| HC_4 | | 3.17E+12 |  | HC_4 | 8.21E+12 |
| HC_5 | | 4.11E+11 |  | HC_5 | 2.69E+12 |
| HC_6 | | 3.75E+11 |  | HC_6 | 2.63E+12 |
| HC_7 | | 2.61E+12 |  | HC_7 | 3.37E+12 |
| HC_8 | | 2.82E+12 |  | HC_8 | 4.87E+12 |
| HC_9 | | 3.49E+12 |  | HC_9 | 9.09E+12 |
| HC_10 | | 4.86E+11 |  | HC_10 | 5.90E+12 |
| HC_11 | | 1.84E+12 |  | HC_11 | 4.58E+12 |
| E_1 | | 1.08E+12 |  | E_1 | 4.09E+12 |
| E_2 | | 1.18E+12 |  | E_2 | 2.92E+12 |
| E_3 | | 9.98E+11 |  | E_3 | 3.63E+12 |
| E_4 | | 1.17E+12 |  | E_4 | 3.99E+12 |
| E_5 | | 9.33E+11 |  | E_5 | 3.48E+12 |
| E_6 | | 1.31E+12 |  | E_6 | 3.23E+12 |
| E_7 | | 1.04E+12 |  | E_7 | 3.98E+12 |
| E_8 | | 1.09E+12 |  | E_8 | 4.20E+12 |
| E_9 | | 1.14E+12 |  | E_9 | 3.02E+12 |
| E_10 | | 1.23E+12 |  | E_10 | 3.52E+12 |
| E_11 | | 8.80E+11 |  | E_11 | 3.46E+12 |
| E_12 | | 1.21E+12 |  | E_12 | 2.39E+12 |
| E_13 | | 9.68E+11 |  | E_13 | 3.48E+12 |
| E_14 | | 1.16E+12 |  | E_14 | 2.69E+12 |
| E_15 | | 1.32E+12 |  | E_15 | 3.50E+12 |
| E_16 | | 8.79E+11 |  | E_16 | 2.87E+12 |
| ES_1 | | 1.70E+12 |  | ES_1 | 3.03E+12 |
| ES_2 | | 7.31E+11 |  | ES_2 | 3.99E+12 |
| ES_3 | | 9.39E+11 |  | ES_3 | 3.30E+12 |
| ES_4 | | 1.63E+12 |  | ES_4 | 3.11E+12 |
| ES_5 | | 3.05E+12 |  | ES_5 | 4.39E+12 |
| ES_6 | | 2.05E+12 |  | ES_6 | 2.68E+12 |
| ES_7 | | 2.70E+12 |  | ES_7 | 3.05E+12 |
| ES_8 | | 1.49E+12 |  | ES_8 | 2.50E+12 |
| ES_9 | | 1.80E+12 |  | ES_9 | 1.37E+12 |
| ES_10 | | 1.19E+12 |  | ES_10 | 3.15E+12 |
| ES_11 | | 1.71E+12 |  | ES_11 | 2.52E+12 |
| ES_12 | | 2.37E+12 |  | ES_12 | 1.56E+12 |
| ES_13 | | 1.31E+12 |  | ES_13 | 2.56E+11 |
| ES_14 | | 1.11E+12 |  | ES_14 | 2.97E+12 |

| **Microsomal fraction** | | |  | **Cytosolic fraction** | | |
| --- | --- | --- | --- | --- | --- | --- |
| **Sample** | **PIP (%)** | **PIPr (%)** |  | **Sample** | **PIP (%)** | **PIPr (%)** |
| HC_4 | 69 | 83 |  | HC_3 | 65 | 83 |
| HC_6 | 67 | 81 |  | HC_5 | 67 | 84 |
| HC_8 | 67 | 82 |  | HC_9 | 65 | 85 |
| E_3 | 65 | 81 |  | E_2 | 68 | 84 |
| E_6 | 70 | 83 |  | E_4 | 68 | 83 |
| E_13 | 69 | 82 |  | E_9 | 64 | 83 |
| E_15 | 66 | 83 |  | E_10 | 65 | 84 |
| ES_5 | 68 | 82 |  | ES_3 | 67 | 84 |
| ES_7 | 65 | 80 |  | ES_8 | 59 | 79 |
| ES_12 | 63 | 77 |  | ES_13 | 51 | 74 |

**Table S3.** Percentage identical peptides (PIP) and percentage identical protein (PIPr) values of technical replicates. (HC= Healthy Controls, E= Early-Stage ADPKD, ES=End Stage-ADPKD)

**Table S4.** Percentage identical peptides (PIP) and percentage identical protein (PIPr) of microsomal fractions. (HC= Healthy Controls, E= Early-Stage ADPKD, ES=End Stage-ADPKD)

|  | **HC1** | **HC2** | **HC3** | **HC4** | **HC5** | **HC6** | **HC7** | **HC8** | **HC9** | **HC10** | **HC11** | **E_1** | **E_2** | **E_3** | **E_4** | **E_5** | **E_6** | **E_7** | **E_8** | **E_9** | **E_10** | **E_11** | **E_12** | **E_13** | **E_14** | **E_15** | **E_16** | **ES_1** | **ES_2** | **ES_3** | **ES_4** | **ES_5** | **ES_6** | **ES_7** | **ES_8** | **ES_9** | **ES_10** | **ES_11** | **ES_12** | **ES_13** | **ES_14** |
| --- | --- | --- | --- | --- | --- | --- | --- | --- | --- | --- | --- | --- | --- | --- | --- | --- | --- | --- | --- | --- | --- | --- | --- | --- | --- | --- | --- | --- | --- | --- | --- | --- | --- | --- | --- | --- | --- | --- | --- | --- | --- |
| **HC1** | x | 46 | 44 | 53 | 45 | 47 | 46 | 49 | 54 | 36 | 37 | 42 | 44 | 44 | 43 | 45 | 45 | 41 | 45 | 43 | 41 | 42 | 42 | 43 | 43 | 43 | 43 | 32 | 41 | 37 | 32 | 50 | 37 | 42 | 26 | 28 | 30 | 29 | 24 | 30 | 25 |
| **HC2** | 66 | x | 55 | 48 | 59 | 61 | 40 | 43 | 46 | 43 | 33 | 52 | 50 | 50 | 47 | 52 | 47 | 51 | 50 | 50 | 48 | 48 | 43 | 54 | 45 | 46 | 46 | 26 | 36 | 30 | 26 | 44 | 29 | 36 | 22 | 23 | 24 | 24 | 19 | 27 | 21 |
| **HC3** | 67 | 72 | x | 47 | 57 | 58 | 39 | 47 | 46 | 48 | 32 | 50 | 54 | 53 | 44 | 53 | 45 | 53 | 50 | 52 | 50 | 52 | 41 | 55 | 43 | 44 | 44 | 27 | 34 | 29 | 27 | 43 | 30 | 36 | 22 | 23 | 24 | 26 | 21 | 26 | 23 |
| **HC4** | 66 | 66 | 66 | x | 49 | 53 | 46 | 59 | 62 | 40 | 39 | 50 | 47 | 51 | 48 | 49 | 50 | 46 | 48 | 47 | 47 | 45 | 45 | 52 | 46 | 50 | 50 | 29 | 37 | 32 | 29 | 57 | 32 | 42 | 22 | 25 | 26 | 26 | 20 | 28 | 22 |
| **HC5** | 67 | 74 | 74 | 67 | x | 62 | 40 | 45 | 47 | 46 | 33 | 51 | 52 | 52 | 46 | 53 | 47 | 52 | 51 | 53 | 50 | 49 | 44 | 56 | 45 | 47 | 47 | 27 | 36 | 30 | 27 | 46 | 29 | 36 | 22 | 23 | 25 | 25 | 20 | 26 | 22 |
| **HC6** | 68 | 74 | 72 | 71 | 74 | x | 40 | 46 | 49 | 46 | 34 | 55 | 53 | 56 | 49 | 55 | 51 | 53 | 52 | 54 | 49 | 50 | 45 | 58 | 46 | 51 | 51 | 27 | 35 | 31 | 27 | 48 | 39 | 37 | 22 | 23 | 25 | 25 | 19 | 27 | 21 |
| **HC7** | 68 | 63 | 63 | 66 | 64 | 65 | x | 42 | 48 | 42 | 29 | 38 | 40 | 39 | 39 | 40 | 42 | 39 | 43 | 39 | 38 | 38 | 39 | 40 | 40 | 39 | 39 | 31 | 37 | 35 | 32 | 44 | 34 | 40 | 26 | 27 | 29 | 28 | 23 | 30 | 24 |
| **HC8** | 65 | 62 | 66 | 71 | 64 | 66 | 63 | x | 54 | 39 | 38 | 44 | 45 | 50 | 44 | 45 | 45 | 44 | 44 | 44 | 45 | 44 | 44 | 47 | 45 | 46 | 46 | 31 | 39 | 31 | 30 | 53 | 32 | 43 | 24 | 25 | 27 | 28 | 24 | 28 | 25 |
| **HC9** | 70 | 67 | 68 | 75 | 68 | 71 | 70 | 68 | x | 39 | 37 | 46 | 46 | 49 | 46 | 48 | 48 | 45 | 48 | 46 | 47 | 45 | 44 | 48 | 45 | 47 | 47 | 32 | 40 | 35 | 32 | 55 | 36 | 45 | 25 | 27 | 28 | 28 | 23 | 29 | 24 |
| **HC10** | 65 | 69 | 72 | 66 | 71 | 71 | 65 | 66 | 68 | x | 25 | 41 | 43 | 44 | 38 | 43 | 39 | 44 | 42 | 42 | 41 | 42 | 36 | 45 | 37 | 38 | 38 | 25 | 31 | 26 | 24 | 38 | 26 | 32 | 20 | 21 | 22 | 23 | 20 | 24 | 20 |
| **HC11** | 65 | 64 | 66 | 67 | 65 | 66 | 61 | 68 | 66 | 65 | x | 31 | 32 | 33 | 32 | 32 | 32 | 30 | 32 | 31 | 31 | 31 | 31 | 32 | 31 | 32 | 32 | 24 | 29 | 24 | 23 | 37 | 25 | 29 | 19 | 20 | 21 | 22 | 17 | 23 | 18 |
| **E_1** | 63 | 72 | 69 | 69 | 70 | 73 | 63 | 63 | 68 | 68 | 64 | x | 53 | 56 | 45 | 61 | 46 | 53 | 55 | 55 | 51 | 50 | 41 | 60 | 42 | 46 | 46 | 26 | 33 | 28 | 25 | 44 | 28 | 35 | 21 | 22 | 23 | 24 | 19 | 25 | 21 |
| **E_2** | 67 | 69 | 71 | 65 | 70 | 70 | 66 | 64 | 68 | 70 | 65 | 69 | x | 57 | 44 | 57 | 45 | 53 | 53 | 54 | 52 | 54 | 41 | 57 | 42 | 43 | 43 | 27 | 35 | 30 | 27 | 47 | 30 | 36 | 22 | 23 | 24 | 25 | 21 | 26 | 22 |
| **E_3** | 65 | 68 | 70 | 71 | 70 | 72 | 66 | 69 | 71 | 71 | 67 | 70 | 73 | x | 47 | 59 | 49 | 55 | 56 | 56 | 56 | 57 | 47 | 62 | 47 | 49 | 49 | 29 | 36 | 31 | 29 | 48 | 31 | 40 | 23 | 25 | 26 | 27 | 22 | 28 | 23 |
| **E_4** | 66 | 67 | 67 | 72 | 68 | 71 | 66 | 66 | 70 | 67 | 65 | 70 | 67 | 70 | x | 46 | 66 | 43 | 44 | 45 | 42 | 43 | 55 | 48 | 57 | 64 | 64 | 26 | 34 | 30 | 26 | 45 | 29 | 36 | 21 | 22 | 24 | 25 | 19 | 27 | 21 |
| **E_5** | 66 | 71 | 71 | 68 | 72 | 72 | 65 | 63 | 70 | 71 | 66 | 75 | 72 | 72 | 69 | x | 47 | 55 | 59 | 58 | 54 | 55 | 44 | 59 | 44 | 46 | 46 | 30 | 37 | 32 | 29 | 45 | 32 | 39 | 24 | 26 | 27 | 27 | 23 | 28 | 24 |
| **E_6** | 65 | 64 | 63 | 72 | 64 | 70 | 68 | 64 | 69 | 64 | 62 | 67 | 65 | 69 | 77 | 67 | x | 45 | 46 | 46 | 43 | 44 | 57 | 50 | 58 | 66 | 66 | 28 | 34 | 31 | 28 | 48 | 31 | 38 | 22 | 23 | 25 | 26 | 19 | 29 | 21 |
| **E_7** | 65 | 70 | 71 | 66 | 69 | 71 | 64 | 65 | 68 | 69 | 65 | 71 | 70 | 72 | 67 | 72 | 65 | x | 54 | 52 | 53 | 51 | 43 | 60 | 44 | 44 | 44 | 28 | 36 | 30 | 28 | 42 | 30 | 38 | 23 | 25 | 25 | 27 | 22 | 28 | 23 |
| **E_8** | 66 | 70 | 69 | 66 | 68 | 70 | 69 | 63 | 69 | 68 | 64 | 71 | 71 | 71 | 68 | 74 | 67 | 73 | x | 55 | 56 | 53 | 42 | 55 | 43 | 45 | 45 | 30 | 37 | 33 | 30 | 45 | 33 | 39 | 24 | 26 | 27 | 28 | 23 | 29 | 24 |
| **E_9** | 68 | 70 | 71 | 67 | 72 | 73 | 66 | 64 | 69 | 69 | 64 | 71 | 72 | 70 | 68 | 74 | 66 | 69 | 73 | x | 52 | 55 | 41 | 56 | 41 | 46 | 46 | 26 | 33 | 29 | 26 | 43 | 29 | 35 | 20 | 22 | 23 | 24 | 19 | 25 | 20 |
| **E_10** | 64 | 70 | 70 | 66 | 70 | 69 | 63 | 65 | 69 | 69 | 64 | 69 | 70 | 72 | 66 | 71 | 63 | 72 | 71 | 70 | x | 52 | 42 | 56 | 43 | 43 | 43 | 28 | 35 | 29 | 27 | 44 | 29 | 37 | 23 | 23 | 24 | 26 | 21 | 27 | 22 |
| **E_11** | 65 | 68 | 69 | 65 | 68 | 69 | 65 | 64 | 68 | 69 | 64 | 66 | 71 | 71 | 67 | 71 | 66 | 70 | 71 | 69 | 70 | x | 42 | 55 | 42 | 43 | 43 | 28 | 36 | 31 | 28 | 43 | 31 | 37 | 23 | 24 | 26 | 27 | 21 | 28 | 23 |
| **E_12** | 63 | 62 | 62 | 66 | 63 | 65 | 63 | 66 | 66 | 65 | 63 | 62 | 63 | 69 | 69 | 65 | 69 | 64 | 65 | 63 | 64 | 66 | x | 46 | 64 | 57 | 57 | 33 | 40 | 35 | 33 | 44 | 33 | 43 | 27 | 28 | 30 | 30 | 25 | 32 | 25 |
| **E_13** | 64 | 70 | 70 | 72 | 70 | 73 | 66 | 67 | 70 | 69 | 65 | 73 | 72 | 75 | 72 | 72 | 71 | 74 | 70 | 71 | 72 | 71 | 67 | x | 47 | 49 | 49 | 27 | 35 | 29 | 26 | 48 | 29 | 37 | 21 | 23 | 24 | 25 | 20 | 27 | 21 |
| **E_14** | 64 | 64 | 64 | 67 | 65 | 67 | 65 | 67 | 68 | 67 | 63 | 62 | 65 | 71 | 71 | 65 | 71 | 67 | 66 | 64 | 66 | 66 | 76 | 70 | x | 58 | 58 | 31 | 39 | 33 | 31 | 44 | 32 | 41 | 26 | 27 | 28 | 29 | 24 | 31 | 24 |
| **E_15** | 64 | 65 | 64 | 71 | 66 | 72 | 66 | 66 | 70 | 65 | 63 | 67 | 64 | 70 | 77 | 68 | 79 | 65 | 66 | 68 | 63 | 65 | 70 | 70 | 72 | x | 65 | 27 | 34 | 30 | 27 | 47 | 29 | 38 | 21 | 22 | 24 | 26 | 19 | 28 | 20 |
| **E_16** | 66 | 68 | 68 | 66 | 68 | 69 | 65 | 67 | 69 | 70 | 65 | 68 | 69 | 72 | 66 | 71 | 65 | 74 | 73 | 68 | 71 | 71 | 68 | 71 | 68 | 65 | x | 31 | 39 | 33 | 31 | 43 | 33 | 41 | 25 | 27 | 28 | 29 | 24 | 30 | 25 |
| **ES_1** | 49 | 45 | 47 | 45 | 47 | 45 | 49 | 48 | 48 | 50 | 47 | 45 | 47 | 49 | 45 | 50 | 45 | 48 | 49 | 46 | 48 | 50 | 53 | 46 | 50 | 45 | 52 | x | 43 | 48 | 61 | 29 | 45 | 50 | 45 | 50 | 54 | 42 | 39 | 43 | 39 |
| **ES_2** | 57 | 55 | 57 | 53 | 57 | 54 | 57 | 56 | 58 | 60 | 57 | 53 | 57 | 59 | 54 | 60 | 51 | 58 | 58 | 55 | 59 | 60 | 60 | 55 | 59 | 52 | 63 | 64 | x | 47 | 43 | 37 | 40 | 48 | 36 | 38 | 41 | 35 | 33 | 36 | 33 |
| **ES_3** | 52 | 47 | 48 | 47 | 49 | 48 | 53 | 47 | 51 | 49 | 47 | 46 | 49 | 49 | 47 | 51 | 47 | 48 | 50 | 49 | 48 | 51 | 52 | 46 | 49 | 46 | 51 | 66 | 59 | x | 54 | 32 | 50 | 48 | 41 | 46 | 48 | 38 | 36 | 40 | 36 |
| **ES_4** | 48 | 43 | 45 | 44 | 45 | 43 | 48 | 46 | 47 | 46 | 45 | 42 | 45 | 46 | 44 | 47 | 44 | 45 | 47 | 44 | 45 | 49 | 51 | 44 | 49 | 44 | 49 | 73 | 60 | 69 | x | 30 | 46 | 50 | 44 | 48 | 53 | 40 | 37 | 42 | 38 |
| **ES_5** | 65 | 59 | 62 | 71 | 62 | 65 | 66 | 69 | 69 | 62 | 63 | 62 | 65 | 68 | 67 | 63 | 69 | 62 | 62 | 63 | 61 | 63 | 64 | 68 | 66 | 68 | 62 | 44 | 51 | 44 | 43 | x | 33 | 42 | 22 | 23 | 25 | 26 | 19 | 28 | 21 |
| **ES_6** | 53 | 49 | 50 | 49 | 50 | 49 | 54 | 49 | 53 | 51 | 49 | 48 | 51 | 50 | 49 | 53 | 49 | 50 | 52 | 50 | 50 | 53 | 54 | 49 | 52 | 48 | 54 | 62 | 59 | 67 | 63 | 47 | x | 51 | 41 | 45 | 45 | 39 | 34 | 39 | 34 |
| **ES_7** | 59 | 55 | 56 | 58 | 56 | 56 | 59 | 58 | 61 | 58 | 55 | 54 | 56 | 59 | 56 | 59 | 57 | 57 | 59 | 56 | 58 | 59 | 63 | 57 | 61 | 57 | 60 | 63 | 63 | 61 | 62 | 55 | 64 | x | 39 | 43 | 46 | 39 | 35 | 41 | 35 |
| **ES_8** | 38 | 37 | 38 | 34 | 37 | 36 | 40 | 38 | 38 | 40 | 37 | 36 | 38 | 38 | 36 | 40 | 34 | 38 | 39 | 36 | 38 | 40 | 41 | 35 | 39 | 34 | 40 | 56 | 50 | 54 | 54 | 33 | 54 | 48 | x | 53 | 52 | 41 | 44 | 38 | 45 |
| **ES_9** | 41 | 38 | 40 | 36 | 39 | 37 | 41 | 39 | 40 | 41 | 39 | 37 | 39 | 39 | 37 | 42 | 36 | 40 | 41 | 38 | 40 | 41 | 42 | 37 | 40 | 36 | 43 | 60 | 55 | 60 | 58 | 34 | 59 | 52 | 65 | x | 58 | 44 | 46 | 42 | 41 |
| **ES_10** | 44 | 41 | 42 | 39 | 42 | 40 | 45 | 42 | 43 | 44 | 41 | 39 | 42 | 42 | 39 | 43 | 39 | 42 | 43 | 40 | 43 | 45 | 46 | 40 | 44 | 39 | 45 | 66 | 58 | 62 | 65 | 38 | 61 | 58 | 63 | 69 | x | 42 | 42 | 43 | 41 |
| **ES_11** | 48 | 45 | 47 | 43 | 46 | 44 | 48 | 47 | 47 | 48 | 45 | 43 | 46 | 47 | 45 | 48 | 44 | 47 | 48 | 45 | 46 | 48 | 51 | 44 | 48 | 44 | 50 | 65 | 59 | 61 | 61 | 41 | 61 | 59 | 59 | 63 | 63 | x | 38 | 51 | 35 |
| **ES_12** | 34 | 32 | 34 | 30 | 34 | 31 | 34 | 33 | 33 | 36 | 32 | 31 | 33 | 33 | 31 | 35 | 29 | 34 | 35 | 32 | 34 | 35 | 36 | 31 | 34 | 30 | 36 | 51 | 47 | 48 | 48 | 28 | 48 | 42 | 61 | 61 | 57 | 53 | x | 32 | 42 |
| **ES_13** | 50 | 46 | 48 | 47 | 47 | 47 | 50 | 49 | 50 | 49 | 49 | 46 | 48 | 50 | 47 | 50 | 48 | 50 | 50 | 47 | 48 | 52 | 55 | 48 | 52 | 47 | 53 | 67 | 60 | 60 | 63 | 46 | 59 | 62 | 51 | 55 | 59 | 66 | 45 | x | 31 |
| **ES_14** | 37 | 36 | 38 | 33 | 37 | 35 | 38 | 37 | 36 | 39 | 36 | 35 | 37 | 37 | 34 | 40 | 33 | 38 | 38 | 36 | 38 | 39 | 39 | 34 | 38 | 33 | 40 | 53 | 47 | 49 | 50 | 31 | 50 | 46 | 62 | 58 | 57 | 53 | 58 | 46 | x |

|  | **HC1** | **HC2** | **HC3** | **HC4** | **HC5** | **HC6** | **HC7** | **HC8** | **HC9** | **HC10** | **HC11** | **E_1** | **E_2** | **E_3** | **E_4** | **E_5** | **E_6** | **E_7** | **E_8** | **E_9** | **E_10** | **E_11** | **E_12** | **E_13** | **E_14** | **E_15** | **E_16** | **ES_1** | **ES_2** | **ES_3** | **ES_4** | **ES_5** | **ES_6** | **ES_7** | **ES_8** | **ES_9** | **ES_10** | **ES_11** | **ES_12** | **ES_13** | **ES_14** |
| --- | --- | --- | --- | --- | --- | --- | --- | --- | --- | --- | --- | --- | --- | --- | --- | --- | --- | --- | --- | --- | --- | --- | --- | --- | --- | --- | --- | --- | --- | --- | --- | --- | --- | --- | --- | --- | --- | --- | --- | --- | --- |
| **HC1** | x | 39 | 43 | 53 | 42 | 39 | 40 | 54 | 54 | 41 | 55 | 40 | 42 | 43 | 45 | 41 | 42 | 42 | 43 | 44 | 42 | 43 | 40 | 41 | 39 | 38 | 39 | 33 | 42 | 38 | 35 | 46 | 39 | 37 | 27 | 25 | 31 | 29 | 23 | 24 | 22 |
| **HC2** | 69 | x | 55 | 40 | 60 | 60 | 52 | 38 | 39 | 52 | 39 | 46 | 54 | 51 | 58 | 54 | 56 | 50 | 50 | 53 | 56 | 50 | 48 | 57 | 47 | 54 | 50 | 30 | 38 | 32 | 31 | 42 | 33 | 32 | 24 | 22 | 27 | 26 | 20 | 24 | 19 |
| **HC3** | 69 | 76 | x | 42 | 62 | 55 | 56 | 44 | 43 | 58 | 43 | 46 | 59 | 52 | 59 | 55 | 56 | 55 | 51 | 52 | 61 | 52 | 51 | 56 | 54 | 54 | 56 | 33 | 41 | 35 | 34 | 44 | 36 | 37 | 27 | 25 | 30 | 29 | 24 | 25 | 22 |
| **HC4** | 74 | 69 | 69 | x | 44 | 41 | 38 | 51 | 61 | 42 | 56 | 43 | 41 | 43 | 46 | 42 | 43 | 42 | 44 | 46 | 44 | 42 | 38 | 42 | 38 | 39 | 39 | 30 | 39 | 35 | 32 | 47 | 35 | 35 | 24 | 22 | 27 | 26 | 20 | 22 | 19 |
| **HC5** | 69 | 77 | 77 | 72 | x | 63 | 54 | 42 | 45 | 56 | 43 | 49 | 59 | 52 | 61 | 56 | 58 | 53 | 53 | 57 | 65 | 53 | 49 | 57 | 49 | 57 | 54 | 31 | 40 | 35 | 33 | 45 | 36 | 35 | 25 | 23 | 28 | 27 | 21 | 24 | 20 |
| **HC6** | 68 | 78 | 75 | 71 | 77 | x | 52 | 38 | 40 | 53 | 39 | 48 | 54 | 50 | 58 | 54 | 57 | 49 | 50 | 52 | 58 | 49 | 46 | 53 | 46 | 57 | 50 | 29 | 38 | 32 | 30 | 42 | 33 | 33 | 23 | 22 | 26 | 25 | 20 | 23 | 19 |
| **HC7** | 68 | 73 | 74 | 66 | 72 | 72 | x | 40 | 39 | 57 | 37 | 42 | 55 | 48 | 52 | 52 | 53 | 51 | 52 | 47 | 55 | 47 | 49 | 50 | 50 | 51 | 53 | 36 | 42 | 38 | 37 | 41 | 38 | 38 | 30 | 27 | 33 | 32 | 25 | 27 | 25 |
| **HC8** | 69 | 67 | 75 | 68 | 70 | 67 | 69 | x | 52 | 42 | 55 | 40 | 41 | 44 | 44 | 42 | 42 | 45 | 42 | 43 | 44 | 43 | 42 | 42 | 43 | 39 | 41 | 35 | 42 | 35 | 35 | 44 | 37 | 39 | 30 | 27 | 31 | 32 | 26 | 26 | 25 |
| **HC9** | 75 | 68 | 67 | 78 | 70 | 67 | 66 | 67 | x | 43 | 57 | 43 | 42 | 43 | 48 | 43 | 43 | 43 | 45 | 48 | 45 | 44 | 38 | 42 | 37 | 39 | 40 | 32 | 42 | 39 | 34 | 49 | 38 | 37 | 26 | 23 | 29 | 27 | 21 | 22 | 20 |
| **HC10** | 68 | 74 | 78 | 67 | 74 | 74 | 75 | 71 | 67 | x | 40 | 45 | 57 | 51 | 57 | 53 | 53 | 52 | 52 | 48 | 58 | 49 | 48 | 53 | 49 | 53 | 52 | 33 | 41 | 35 | 33 | 44 | 36 | 36 | 27 | 25 | 30 | 30 | 23 | 24 | 23 |
| **HC11** | 76 | 70 | 70 | 78 | 72 | 70 | 66 | 69 | 78 | 69 | x | 41 | 41 | 42 | 45 | 41 | 41 | 42 | 42 | 46 | 43 | 42 | 38 | 41 | 37 | 38 | 38 | 29 | 38 | 34 | 31 | 46 | 35 | 35 | 24 | 22 | 27 | 26 | 21 | 22 | 19 |
| **E_1** | 62 | 69 | 67 | 65 | 68 | 70 | 65 | 64 | 63 | 66 | 65 | x | 45 | 53 | 53 | 52 | 52 | 49 | 53 | 55 | 48 | 49 | 44 | 48 | 43 | 46 | 43 | 28 | 36 | 32 | 29 | 40 | 31 | 33 | 24 | 22 | 26 | 25 | 20 | 22 | 19 |
| **E_2** | 70 | 73 | 76 | 70 | 75 | 73 | 73 | 69 | 70 | 76 | 71 | 65 | x | 50 | 58 | 53 | 56 | 51 | 50 | 50 | 62 | 51 | 48 | 53 | 48 | 55 | 55 | 33 | 41 | 36 | 34 | 48 | 38 | 36 | 26 | 24 | 30 | 29 | 22 | 24 | 22 |
| **E_3** | 67 | 74 | 75 | 67 | 73 | 73 | 72 | 70 | 66 | 73 | 67 | 70 | 72 | x | 57 | 55 | 55 | 55 | 57 | 57 | 54 | 59 | 54 | 57 | 51 | 48 | 49 | 34 | 41 | 34 | 34 | 43 | 36 | 37 | 28 | 26 | 31 | 30 | 24 | 26 | 23 |
| **E_4** | 71 | 77 | 76 | 73 | 78 | 78 | 73 | 69 | 72 | 76 | 74 | 70 | 77 | 74 | x | 64 | 65 | 58 | 58 | 60 | 61 | 57 | 52 | 64 | 52 | 54 | 51 | 32 | 41 | 36 | 33 | 48 | 37 | 36 | 26 | 24 | 29 | 28 | 22 | 24 | 21 |
| **E_5** | 66 | 75 | 75 | 67 | 75 | 75 | 73 | 69 | 66 | 74 | 68 | 71 | 72 | 75 | 77 | x | 62 | 58 | 56 | 55 | 55 | 54 | 55 | 59 | 56 | 53 | 51 | 34 | 41 | 36 | 34 | 42 | 37 | 38 | 28 | 26 | 31 | 30 | 24 | 26 | 23 |
| **E_6** | 69 | 75 | 74 | 69 | 75 | 76 | 74 | 69 | 68 | 74 | 69 | 72 | 73 | 74 | 78 | 77 | x | 58 | 57 | 56 | 58 | 53 | 51 | 60 | 52 | 53 | 51 | 32 | 40 | 35 | 33 | 44 | 36 | 36 | 27 | 25 | 30 | 29 | 23 | 25 | 22 |
| **E_7** | 64 | 72 | 75 | 64 | 71 | 70 | 73 | 70 | 63 | 73 | 65 | 66 | 69 | 74 | 71 | 73 | 73 | x | 57 | 54 | 57 | 54 | 55 | 58 | 56 | 50 | 54 | 34 | 41 | 34 | 34 | 42 | 36 | 38 | 29 | 26 | 31 | 31 | 25 | 27 | 24 |
| **E_8** | 69 | 75 | 73 | 69 | 74 | 73 | 76 | 68 | 69 | 73 | 70 | 69 | 71 | 76 | 76 | 76 | 76 | 74 | x | 58 | 54 | 57 | 51 | 55 | 49 | 49 | 50 | 33 | 40 | 36 | 34 | 42 | 37 | 37 | 28 | 25 | 31 | 29 | 24 | 25 | 22 |
| **E_9** | 71 | 75 | 72 | 73 | 76 | 74 | 71 | 67 | 73 | 70 | 72 | 70 | 71 | 74 | 77 | 73 | 74 | 71 | 77 | x | 55 | 57 | 48 | 55 | 46 | 50 | 49 | 30 | 39 | 36 | 32 | 45 | 36 | 35 | 24 | 22 | 28 | 26 | 20 | 23 | 19 |
| **E_10** | 68 | 75 | 79 | 69 | 78 | 73 | 74 | 71 | 70 | 76 | 70 | 69 | 77 | 76 | 77 | 75 | 75 | 75 | 75 | 74 | x | 54 | 51 | 58 | 51 | 58 | 58 | 33 | 42 | 36 | 34 | 46 | 36 | 37 | 26 | 24 | 30 | 28 | 22 | 25 | 22 |
| **E_11** | 69 | 73 | 73 | 67 | 72 | 71 | 72 | 69 | 67 | 73 | 68 | 65 | 73 | 77 | 75 | 74 | 73 | 71 | 75 | 73 | 75 | x | 52 | 55 | 49 | 47 | 48 | 33 | 41 | 35 | 34 | 43 | 37 | 36 | 27 | 25 | 31 | 29 | 23 | 26 | 22 |
| **E_12** | 60 | 67 | 70 | 59 | 68 | 65 | 69 | 67 | 59 | 68 | 60 | 63 | 65 | 72 | 67 | 73 | 68 | 72 | 69 | 64 | 69 | 70 | x | 53 | 61 | 47 | 50 | 39 | 44 | 36 | 38 | 39 | 38 | 41 | 34 | 32 | 35 | 36 | 30 | 32 | 28 |
| **E_13** | 67 | 77 | 77 | 67 | 75 | 73 | 71 | 70 | 66 | 75 | 68 | 68 | 73 | 76 | 77 | 74 | 75 | 76 | 74 | 73 | 78 | 75 | 70 | x | 54 | 51 | 50 | 31 | 39 | 32 | 32 | 44 | 34 | 34 | 26 | 24 | 29 | 28 | 22 | 25 | 21 |
| **E_14** | 59 | 66 | 71 | 58 | 65 | 64 | 68 | 67 | 55 | 69 | 58 | 62 | 63 | 70 | 64 | 71 | 65 | 74 | 66 | 62 | 68 | 67 | 76 | 69 | x | 47 | 50 | 38 | 42 | 34 | 36 | 39 | 36 | 40 | 33 | 31 | 34 | 35 | 30 | 31 | 29 |
| **E_15** | 65 | 75 | 74 | 67 | 75 | 76 | 70 | 67 | 64 | 74 | 66 | 68 | 71 | 71 | 74 | 75 | 73 | 71 | 71 | 72 | 74 | 70 | 68 | 73 | 67 | x | 55 | 31 | 38 | 33 | 32 | 41 | 34 | 35 | 25 | 23 | 28 | 27 | 22 | 24 | 20 |
| **E_16** | 66 | 74 | 77 | 65 | 72 | 72 | 75 | 70 | 64 | 75 | 66 | 67 | 72 | 74 | 72 | 75 | 73 | 77 | 74 | 71 | 76 | 73 | 74 | 74 | 74 | 75 | x | 35 | 41 | 35 | 35 | 40 | 37 | 39 | 29 | 27 | 32 | 31 | 25 | 27 | 23 |
| **ES_1** | 52 | 53 | 56 | 49 | 53 | 52 | 58 | 57 | 50 | 55 | 50 | 49 | 52 | 57 | 53 | 57 | 53 | 56 | 55 | 52 | 54 | 56 | 64 | 54 | 60 | 54 | 60 | x | 52 | 50 | 58 | 33 | 51 | 55 | 50 | 47 | 56 | 54 | 42 | 40 | 40 |
| **ES_2** | 65 | 65 | 68 | 63 | 67 | 63 | 68 | 68 | 64 | 68 | 64 | 60 | 65 | 67 | 66 | 68 | 66 | 66 | 66 | 65 | 68 | 68 | 69 | 66 | 65 | 65 | 69 | 69 | x | 52 | 54 | 46 | 52 | 56 | 41 | 36 | 47 | 44 | 33 | 32 | 32 |
| **ES_3** | 61 | 56 | 57 | 59 | 58 | 55 | 60 | 56 | 61 | 57 | 58 | 52 | 58 | 56 | 58 | 58 | 57 | 54 | 58 | 58 | 57 | 59 | 56 | 54 | 52 | 55 | 57 | 64 | 68 | x | 59 | 38 | 58 | 52 | 39 | 35 | 48 | 41 | 30 | 30 | 29 |
| **ES_4** | 57 | 55 | 58 | 54 | 56 | 54 | 60 | 58 | 56 | 57 | 54 | 51 | 56 | 58 | 55 | 58 | 56 | 57 | 57 | 56 | 57 | 59 | 61 | 56 | 57 | 55 | 60 | 74 | 72 | 73 | x | 35 | 54 | 54 | 45 | 42 | 53 | 48 | 37 | 36 | 35 |
| **ES_5** | 72 | 68 | 69 | 72 | 70 | 67 | 66 | 68 | 74 | 70 | 73 | 63 | 73 | 66 | 72 | 65 | 68 | 64 | 67 | 70 | 70 | 67 | 59 | 68 | 58 | 65 | 64 | 49 | 63 | 58 | 54 | x | 40 | 37 | 26 | 23 | 30 | 28 | 21 | 23 | 21 |
| **ES_6** | 63 | 59 | 62 | 59 | 60 | 58 | 63 | 61 | 62 | 60 | 60 | 54 | 61 | 60 | 61 | 61 | 60 | 59 | 62 | 62 | 60 | 63 | 60 | 59 | 57 | 57 | 61 | 66 | 71 | 74 | 71 | 60 | x | 53 | 40 | 37 | 49 | 43 | 33 | 32 | 31 |
| **ES_7** | 57 | 59 | 62 | 55 | 59 | 57 | 63 | 63 | 56 | 61 | 55 | 56 | 57 | 62 | 58 | 64 | 60 | 62 | 60 | 58 | 61 | 61 | 67 | 60 | 65 | 60 | 65 | 73 | 74 | 65 | 71 | 54 | 68 | x | 48 | 42 | 52 | 49 | 38 | 36 | 36 |
| **ES_8** | 37 | 38 | 42 | 35 | 38 | 37 | 44 | 44 | 35 | 42 | 36 | 38 | 38 | 43 | 38 | 43 | 40 | 44 | 40 | 37 | 40 | 41 | 49 | 41 | 50 | 40 | 45 | 59 | 48 | 44 | 51 | 35 | 47 | 56 | x | 54 | 53 | 57 | 51 | 43 | 51 |
| **ES_9** | 36 | 37 | 40 | 34 | 37 | 37 | 42 | 41 | 33 | 41 | 34 | 37 | 37 | 42 | 37 | 42 | 39 | 41 | 39 | 36 | 39 | 40 | 48 | 40 | 49 | 39 | 44 | 58 | 46 | 42 | 50 | 34 | 45 | 53 | 67 | x | 50 | 55 | 52 | 47 | 45 |
| **ES_10** | 49 | 49 | 52 | 46 | 49 | 48 | 54 | 53 | 47 | 52 | 46 | 47 | 49 | 53 | 49 | 53 | 50 | 53 | 51 | 48 | 51 | 53 | 58 | 51 | 56 | 50 | 55 | 70 | 62 | 59 | 67 | 46 | 62 | 69 | 63 | 62 | x | 57 | 42 | 39 | 41 |
| **ES_11** | 42 | 44 | 47 | 40 | 43 | 42 | 49 | 48 | 39 | 47 | 40 | 42 | 44 | 48 | 44 | 49 | 45 | 48 | 46 | 42 | 45 | 47 | 54 | 47 | 56 | 46 | 51 | 67 | 55 | 48 | 56 | 39 | 53 | 62 | 69 | 70 | 68 | x | 49 | 41 | 47 |
| **ES_12** | 32 | 34 | 37 | 30 | 34 | 33 | 38 | 38 | 30 | 37 | 31 | 34 | 34 | 38 | 33 | 38 | 35 | 39 | 35 | 32 | 35 | 36 | 44 | 36 | 46 | 35 | 39 | 51 | 41 | 36 | 44 | 31 | 40 | 47 | 65 | 69 | 54 | 65 | x | 44 | 45 |
| **ES_13** | 36 | 39 | 40 | 34 | 38 | 38 | 43 | 41 | 33 | 40 | 34 | 38 | 38 | 43 | 38 | 43 | 39 | 43 | 40 | 36 | 40 | 41 | 48 | 41 | 49 | 40 | 45 | 55 | 44 | 39 | 47 | 34 | 43 | 50 | 61 | 67 | 56 | 63 | 65 | x | 37 |
| **ES_14** | 31 | 32 | 35 | 29 | 32 | 32 | 37 | 37 | 29 | 36 | 30 | 32 | 33 | 36 | 32 | 36 | 34 | 37 | 33 | 30 | 34 | 34 | 41 | 34 | 43 | 33 | 38 | 49 | 40 | 36 | 42 | 29 | 38 | 46 | 66 | 60 | 51 | 60 | 61 | 59 | x |

**Table S5.** Percentage identical peptides (PIP) and percentage identical protein (PIPr) of cytosolic fractions. (HC= Healthy Controls, E= Early-Stage ADPKD, ES=End Stage-ADPKD)

**
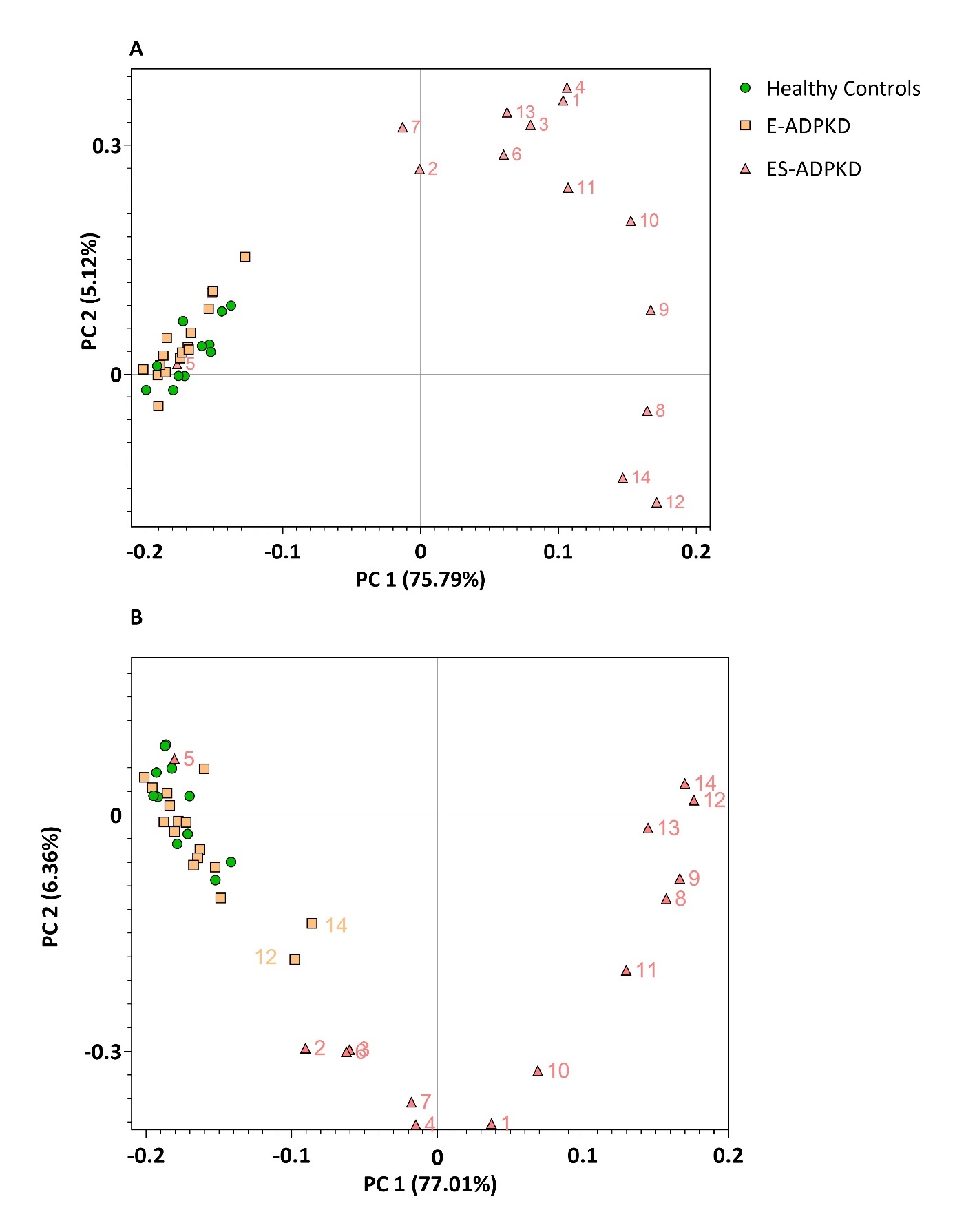
**

**Figure S1.** Principal Component Analysis (PCA) of healthy controls (green), E-ADPKD (orange), and ES‑ADPKD samples (red) in microsomal fraction (**A**) and cytosolic fraction (**B**). The analyses were based on percentage identical protein (PIPr) values.


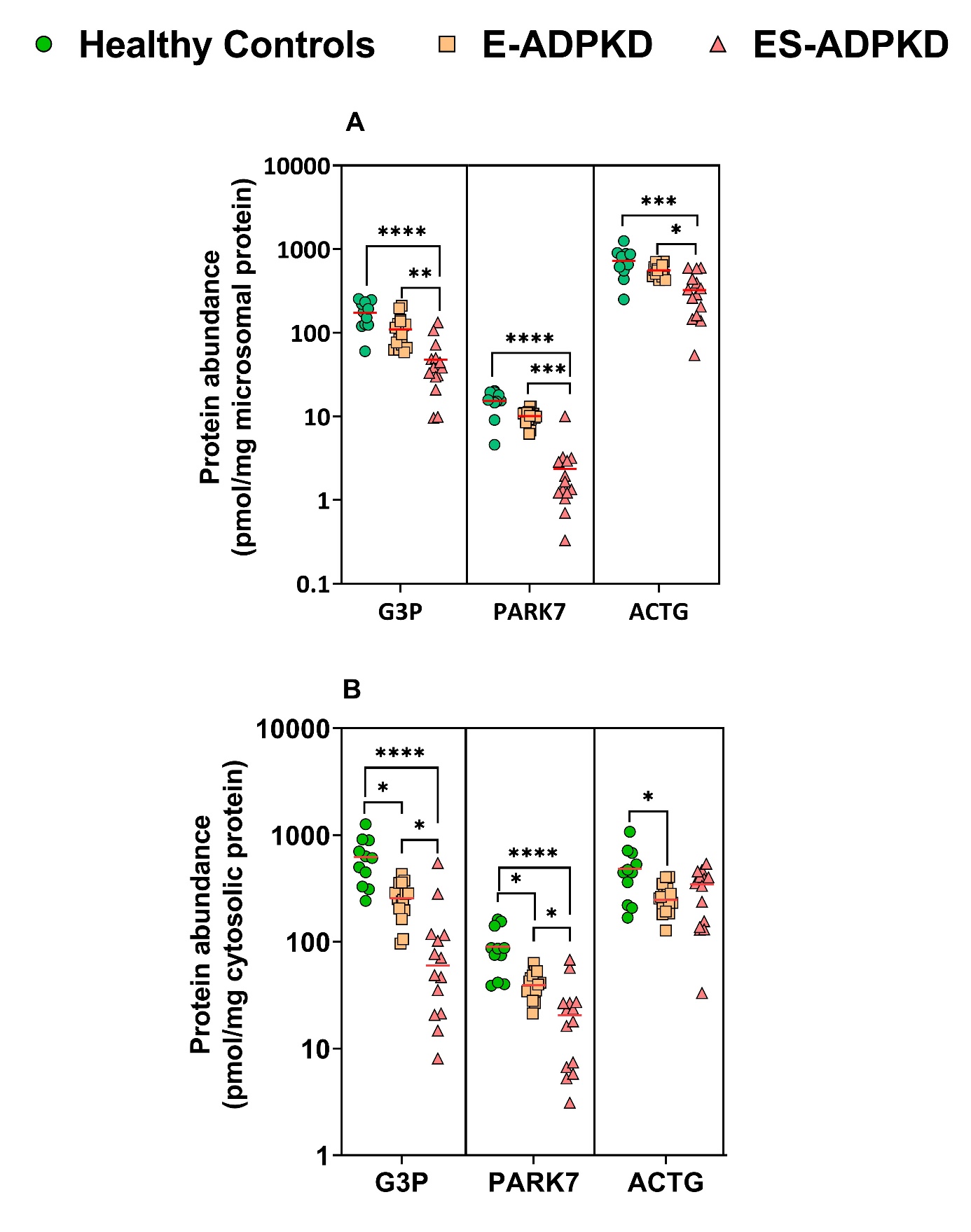


**Figure S2.** Abundances of housekeeping proteins in microsomal (A) and cytosolic fraction (B).


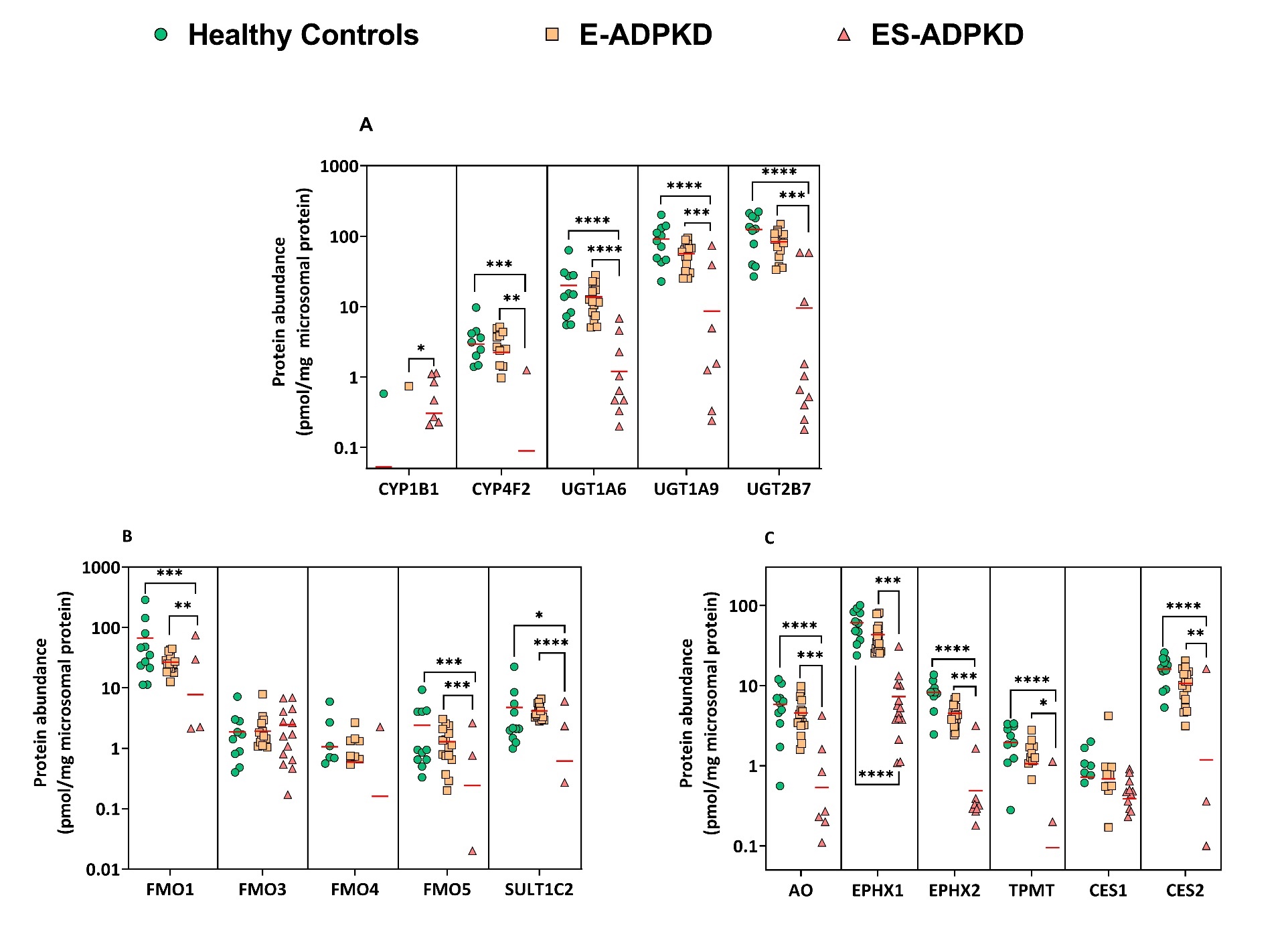


**Figure S3.** Normalized abundance of CYP and UGT enzymes (**A**), FMOs and a SULT (**B**), and oxidases, hydrolases, transferases, and esterases (**C**) in the microsomal fraction of healthy, E-ADPKD and ES-ADPKD kidney tissues, excluding 0 expressions; the lines depict the mean. Differences between the groups were assessed using the Kruskal Wallis and Dunn’s test. (*P < 0.05, **P < 0.01, ***P < 0.001, ****P < 0.0001.)


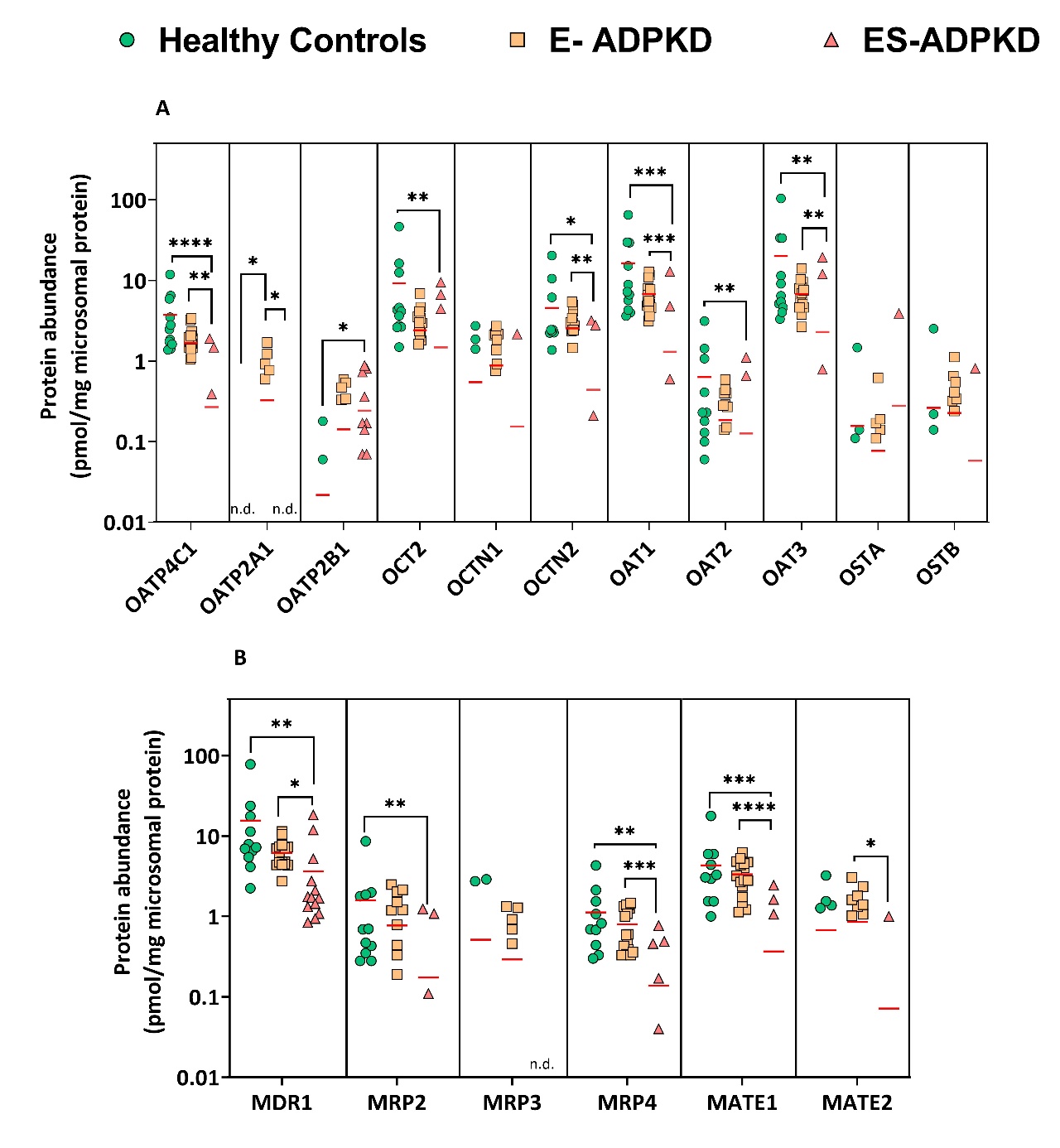


**Figure S4.** Normalized abundance of uptake transporters (**A**) and efflux transporters (**B**) in the microsomal fraction of healthy, E-ADPKD, and ES-ADPKD kidney tissues, excluding 0 expressions; the lines depict the mean. Differences between the groups were assessed using the Kruskal Wallis and Dunn’s test. (*P < 0.05, **P < 0.01, ***P < 0.001, ****P < 0.0001.)


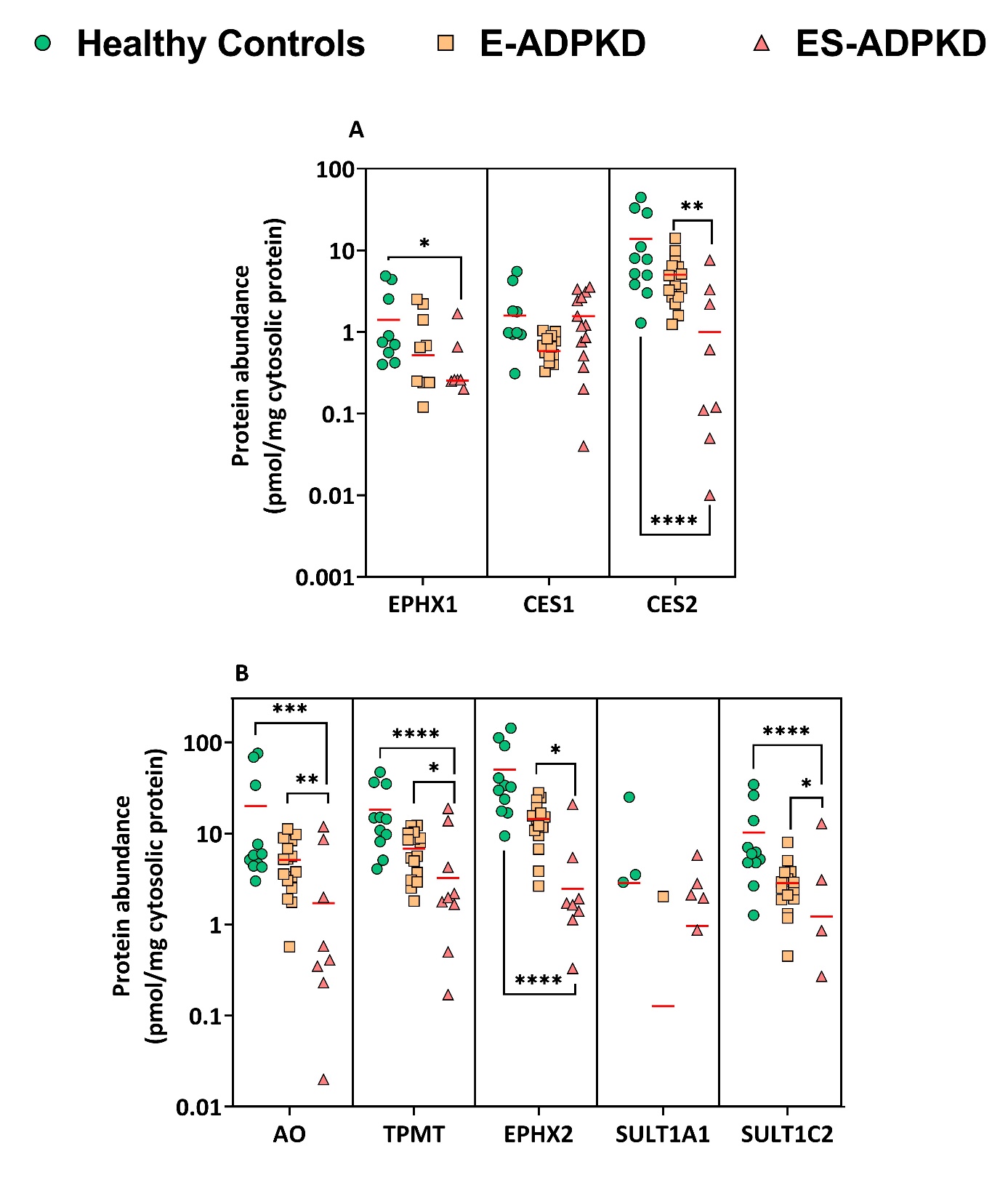


**Figure S5.** Normalized abundance of microsomal enzymes (**A**) and cytosolic enzymes (**B**) in the cytosolic fraction of healthy, E-ADPKD, and ES-ADPKD kidney tissues, excluding 0 expressions; the lines depict the mean. Differences between the groups were assessed using the Kruskal Wallis and Dunn’s test. (*P < 0.05, **P < 0.01, ***P < 0.001, ****P < 0.0001.)

| **Uniprot name** | **Microsome** | | | | | | **Cytosol** | | | | | |
| --- | --- | --- | --- | --- | --- | --- | --- | --- | --- | --- | --- | --- |
|  | **Healthy** | | **E-ADPKD** | | **ES-ADPKD** | | **Healthy** | | **E-ADPKD** | | **ES-ADPKD** | |
|  | **Median** ^a^ | **Rank** | **Median** ^a^ | **Rank** | **Median** ^a^ | **Rank** | **Median** ^b^ | **Rank** | **Median** ^b^ | **Rank** | **Median** ^b^ | **Rank** |
| ACTG | 751.69 | 1 | 560.25 | 1 | 307.9 | 2 | 446.74 | 5 | 246.66 | 3 | 344.38 | 6 |
| POTEF | 386.48 | 2 | 263.15 | 3 | 92.34 | 7 | 102.57 | 58 | 59.71 | 50 | 138.87 | 11 |
| ALBU | 301.68 | 3 | 221.29 | 4 | 388.53 | 1 | 1012.29 | 1 | 491.29 | 1 | 1605.31 | 1 |
| AMPN | 233.38 | 4 | 175.99 | 6 | 3.96 | 236 | 8.87 | 520 | 6.48 | 406 | 0.38 | 1169 |
| ALDOB | 200.05 | 5 | 123.51 | 9 | 0.56 | 924 | 407.89 | 7 | 241.06 | 4 | 0.8 | 889 |
| G3P | 176.68 | 6 | 105.28 | 11 | 38.31 | 21 | 609.39 | 2 | 255.56 | 2 | 59.83 | 23 |
| AT1B1 | 176.25 | 7 | 127.98 | 7 | 3.35 | 289 | BLQ | 1939 | BLQ | 2011 | BLQ | 2172 |
| AT1A1 | 174.29 | 8 | 176.44 | 5 | 8.09 | 125 | BLQ | 1896 | 0.78 | 1333 | BLQ | 1708 |
| HSPB1 | 171.23 | 9 | 124.59 | 8 | 42.93 | 19 | 79.29 | 86 | 60.41 | 48 | 41.19 | 44 |
| LDHB | 165.88 | 10 | 81.79 | 17 | 5.58 | 174 | 313.81 | 12 | 191.61 | 7 | 38.66 | 51 |
| CRYAB | 160.69 | 11 | 275.83 | 2 | 9.38 | 106 | 99.32 | 63 | 103.44 | 22 | 6.21 | 250 |
| ACTBL | 34.65 | 129 | 118.81 | 10 | 8.09 | 126 | 34.77 | 192 | 22.39 | 159 | 27.11 | 72 |
| IGKC | 34.76 | 128 | 37.13 | 67 | 177.98 | 3 | 93.08 | 69 | 60.19 | 49 | 406.63 | 4 |
| IGHG1 | 32.18 | 150 | 16.63 | 264 | 136.18 | 4 | 171.59 | 23 | 70.92 | 37 | 349.45 | 5 |
| HBB | 44.6 | 86 | 28.93 | 113 | 132.29 | 5 | 476.21 | 4 | 117.49 | 16 | 467.28 | 3 |
| HBA | 71.69 | 37 | 56.67 | 32 | 107.96 | 6 | 601.47 | 3 | 102.22 | 23 | 527.54 | 2 |
| CO6A1 | 19.37 | 296 | 19.27 | 221 | 91.27 | 8 | 1.6 | 1333 | 1.13 | 1146 | 5.42 | 279 |
| ACTH | 28.23 | 185 | 25.26 | 137 | 81.44 | 9 | 22.66 | 273 | 14.15 | 234 | 55.29 | 28 |
| IGLL5 | 19.25 | 301 | 24.62 | 145 | 75.32 | 10 | 53.88 | 138 | 23.33 | 151 | 219.76 | 9 |
| PEBP1 | 114.42 | 20 | 68.04 | 23 | 3.34 | 290 | 434.5 | 6 | 199.8 | 5 | 48.42 | 35 |
| ENOA | 132.56 | 14 | 74.73 | 22 | 9.74 | 100 | 398.99 | 8 | 193.67 | 6 | 70.62 | 19 |
| TPIS | 71.2 | 38 | 41.96 | 53 | 4.35 | 212 | 373.27 | 9 | 178.05 | 8 | 72.96 | 18 |
| GATM | 106.12 | 21 | 82.63 | 16 | 1.32 | 611 | 366.62 | 10 | 165.12 | 10 | 2.48 | 466 |
| PGK1 | 53.33 | 61 | 28.81 | 114 | 11.28 | 84 | 325.89 | 11 | 165.37 | 9 | 51.45 | 33 |
| TRFE | 16.72 | 338 | 14.11 | 304 | 27.02 | 34 | 102.1 | 61 | 39.22 | 87 | 289.84 | 7 |
| TAGL | 20.37 | 286 | 17.41 | 250 | 41.97 | 20 | 111.73 | 49 | 92.83 | 26 | 229.15 | 8 |
| A1AG1 | 7.62 | 625 | 6.41 | 588 | 13.6 | 72 | 92.75 | 70 | 49.61 | 61 | 203.91 | 10 |

**Table S6**. The 10 most abundant proteins in the microsomal and cytosolic fraction of healthy controls (HC), Early-stage (E-)ADPKD, and end-stage (ES-) ADPKD. Proteins were ranked based on the median concentrations. (BLQ = Below limit of quantification)

**Figure S6.** Proportional abundances of drug-metabolizing enzymes in the microsomal fraction of healthy control, early-stage ADPKD (E-ADPKD), and end-stage ADPKD (ES-ADPKD) samples.


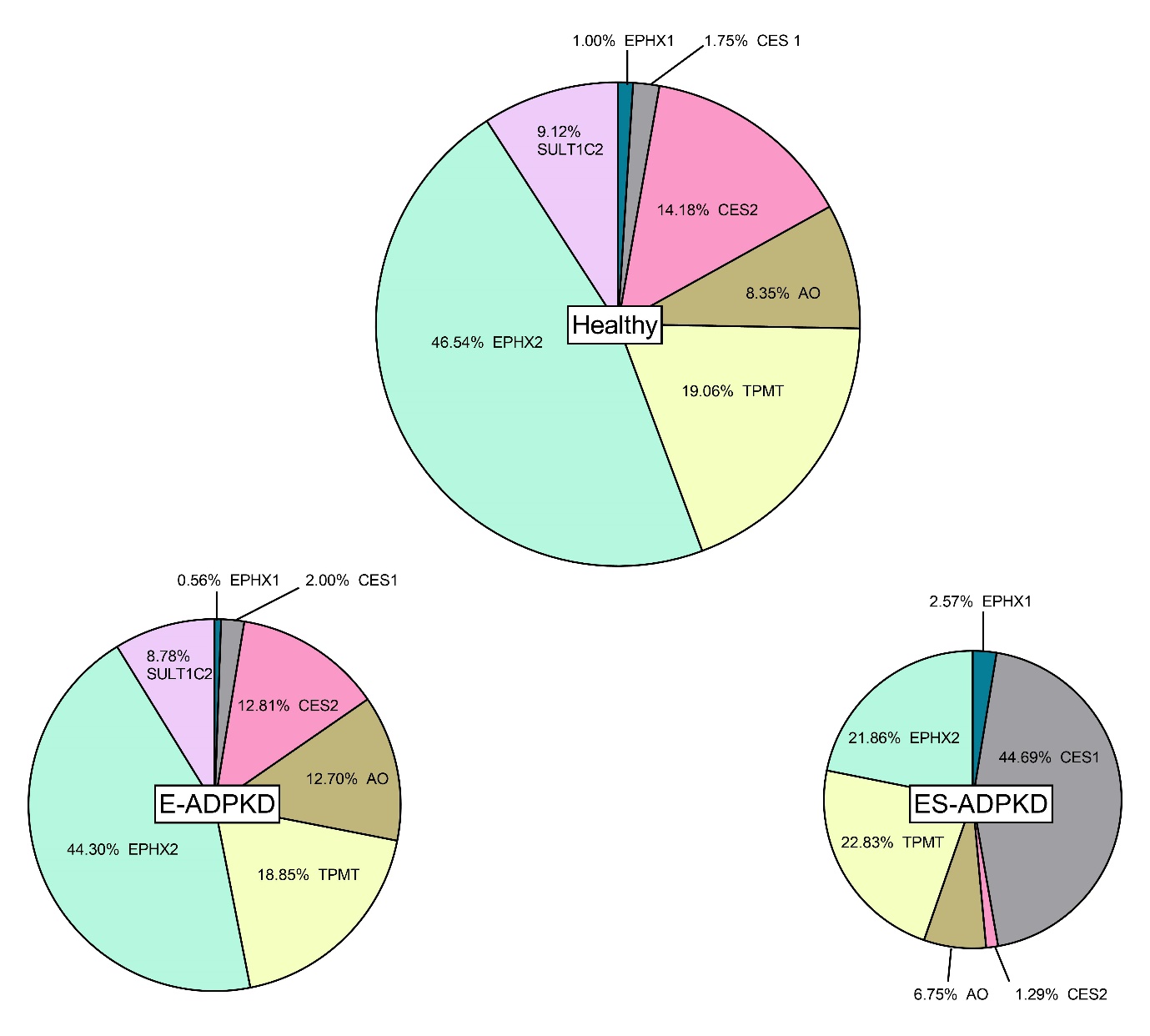


**Figure S7.** Proportional abundances of drug-metabolizing enzymes in the cytosolic fraction of healthy control, early-stage ADPKD (E-ADPKD), and end stage-ADPKD (ES-ADPKD) samples.

**Figure S8.** Proportional abundances of uptake drug transporters (A) and efflux drug transporters (B) in the microsomal fraction of healthy control and Early-stage ADPKD (E-ADPKD) samples.
